# Expression and integrated network analyses revealed functional divergence of NHX-type Na^+^/H^+^ exchanger genes in poplar

**DOI:** 10.1038/s41598-017-02894-8

**Published:** 2017-06-01

**Authors:** Fengxia Tian, Ermei Chang, Yu Li, Pei Sun, Jianjun Hu, Jin Zhang

**Affiliations:** 1 College of Life Science and Technology, Nanyang Normal University, Nanyang, Henan 473061 P.R. China; 2 State Key Laboratory of Tree Genetics and Breeding, Key Laboratory of Tree Breeding and Cultivation of the State Forestry Administration, Research Institute of Forestry, Chinese Academy of Forestry, Beijing, 100091 P.R. China

## Abstract

The Na^+^/H^+^ antiporters (NHXs) are secondary ion transporters to exchange H^+^ and transfer the Na^+^ or K^+^ across membrane, they play crucial roles during plant development and stress responses. To gain insight into the functional divergence of *NHX* genes in poplar, eight *PtNHX* were identified from *Populus trichocarpa* genome. PtNHXs containing 10 transmembrane helices (TMH) and a hydrophilic C-terminal domain, the TMH compose a hollow cylinder to provide the channel for Na^+^ and H^+^ transport. The expression patterns and *cis*-acting elements showed that all the *PtNHXs* were response to single or multiple stresses including drought, heat, cold, salinity, MV, and ABA. Both the co-expression network and protein-protein interaction network of PtNHXs implying their functional divergence. Interestingly, although *PtNHX7* and *PtNHX8* were generated by whole genome duplication event, they showed significant differences in expression pattern, protein structure, co-expressed genes, and interacted proteins. Only PtNHX7 interact with CBL and CIPK, indicating PtNHX7 is the primary NHX involved in CBL-CIPK pathway during salt stress responses. Natural variation analysis based on 549 *P. trichocarpa* individuals indicated the frequency of SNPs in *PtNHX7* was significantly higher than other *PtNHXs*. Our findings provide new insights into the functional divergence of *NHX* genes in poplar.

## Introduction

Ion and pH homeostasis play important regulatory roles in cellular processes controlling plant growth and development. The optimal ion and pH gradients are dependent on H^+^-translocating enzymes (H^+^-pumps) and cation/H^+^ exchangers^1, 2^. Among the numerous transporters in monovalent cation/proton antiporter (CPA1) family, the Na^+^/H^+^ antiporters (NHXs) are secondary ion transporters to exchange H^+^ and transfer the Na^+^ or K^+^ across membrane^3^. To date, all the sequenced eukaryotes containing multiple NHX-like proteins, which were designated as Na^+^/H^+^ exchangers (NHEs), except for yeast only contains a single NHX^4^. Based on their subcellular localization, NHXs are classified into three major classes: plasma membrane (PM)-class, endosomal (Endo)-class, and vacuole (Vac)-class^5^. In mammalian, the specialized subcellular functions of NHEs are dependent on their organelle-specific distribution^6^.

In *Arabidopsis*, NHXs are consisting of six intracellular NHXs (1–6) and two plasma membrane bound NHXs (7 and 8). Among the six intracellular NHXs, four (AtNHX1–4) belong to Vac-class and two (AtNHX5, 6) belong to Endo-class NHX^4^. Based on the biochemical and kinetic analyses, NHEs containing 10–12 transmembrane domains and they may function as homodimers^6^. Although AtNHX1 differ from other known NHX-like antiporters in topological feature, it still contains 10–12 transmembrane domains according to the hydrophobicity analysis^7^.

NHXs involve in various biological processes such as salt stress response^8, 9^, pH homeostasis^10, 11^, K^+^ homeostasis^12, 13^, cell expansion^9, 14^, cellular vesicle trafficking^15, 16^. The *Arabidopsis nhx1 nhx2* double mutant seedlings showed significantly reduced growth compared with either single mutant or wild-type plants. Microscopy observation indicated that cell expansion was reduced in all tissues of *nhx1 nhx2* double mutant, especially in rapidly elongating organs, e.g. flower filaments and etiolated hypocotyls^17^. In addition, the *nhx1 nhx2* double mutant sensitive to external K^+^ and showed curled root, poor expanded and yellow leaves^17, 18^. The Endo-class NHXs (NHX5 and NHX6) localized in the *trans*-Golgi network (TGN) and they played crucial regulatory roles in vesicle trafficking, especially to the vacuole^9^. Under salt stress, the high concentration of extracellular Na^+^ favouring Na^+^ influx into the cell. When the Na^+^ was accumulated to detrimental level, the PM-class NHX (NHX7/SOS1) actively extrude Na^+^ out of the cell, and the Vac-class NHX mediate the sequestration of Na^+^ into vacuole^12, 19, 20^.

Because of the economic importance in pulp and biofuel production^21^ and the completion of whole genome sequence^22^, *Populus* has been to hotspot for gene functional studies in woody species. In previous study, six putative Vac-class NHXs were identified from a high salinity tolerant poplar species, *P. euphratica* ^23^. To reveal the functional divergence of *NHXs* in *P. trichocarpa*, we performed a systematic analysis including phylogenetic relationships, gene structures, conserved protein domains, protein structures, *cis*-acting elements, expression compendium, co-expression network and protein-protein interaction network. Our results provide solid foundation for the study of the evolution and functions of *NHX* genes in poplar.

## Materials and Methods

### Characteristics and phylogenetic analysis of *Populus NHXs*

To identify *Populus NHX* genes, the published *Arabidopsis* and rice NHX protein sequences were searched against the *P. trichocarpa* (Pt) genome (http://phytozome.jgi.doe.gov/pz/portal.html#) through tBLASTn. All homologous protein sequences of the NHX candidates were accepted if they were satisfied with the expectation value (*E*) < 10^–40^. To further confirm the transmembrane helices (TMH) in NHX, the candidate sequences were scanned with TMHMM v.2.0 (http://www.cbs.dtu.dk/services/TMHMM/). Multiple sequences alignment of NHX protein sequences from *P. trichocarpa* and other species were performed using the Clustal X2.1^24^. Phylogenetic tree was constructed using maximum likelihood (ML) method by PhyML V3.0 with 1,000 bootstrap replicates^25^.

### Chromosome location and bioinformatics analyses

The chromosome locations of *Populus NHX* genes were analyzed based on the location sites on *Populus* chromosomes. Location information of *Arabidopsis NHX* genes were obtained from TAIR (https://www.arabidopsis.org/). The gene structure (exon and intron) was showed using Gene Structure Display Server (GSDS, http://gsds.cbi.pku.edu.cn). The duplication events were analyzed based on the synteny blocks from the Plant Genome Duplication Database (PGDD, http://chibba.agtec.uga.edu/duplication/). The conserved motifs were identified using MEME (http://meme-suite.org/tools/meme). For *cis*-acting elements, −1,500 nt upstream to + 500 nt downstream of the transcription start site (TSS) were analyzed by PlantCARE^26^. The PtNHX proteins were modelled using I-TASSER^27^.

### Calculation of *K*a/*K*s

The PAL2NAL program (http://www.bork.embl.de/pal2nal/) was used to estimate synonymous (*K*s) and nonsynonymous (*K*a) substitution rates. The window size 60 bp and 90 bp were used for sliding window analysis. Divergence time (T) was calculated using the formula T = *K*s/2λ (λ = 9.1 × 10^–9^ for *Populus*)^28^.

### Plant material, abiotic stresses, and RT-PCR analysis

Two-month-old *P. trichocarpa* grown at 23–25 °C under long-day condition (16 h/8 h light/dark). Six tissues including shoot apical meristem (SAM), young leaves (YL), mature leaves (ML), primary stems (PS), secondary stems (SS), and roots (R) were collected for RT-PCR. For abiotic stresses or hormone treatment, the seedlings were treated with 10% polyethylene glycol (PEG, for drought stress), 37 °C (for heat stress), 4 °C (for cold stress), 150 mM NaCl (for salt stress), 100 μM methyl viologen (MV, for oxidative stress) or 100 μM abscisic acid (ABA) and the first fully expanded leaves were used for further study. The dosages of the abiotic stresses and hormone treatment were determined based on treatments in poplar^29, 30^. For different tissues and various stresses, three biological replicates were performed.

Total RNA was extracted using the RNeasy Plant Mini Kit (Qiagen), the RNase-free DNase I (Qiagen) was used to remove genomic DNA. First-strand cDNA was synthesized with ~1 μg RNA using the SuperScript III reverse transcription kit (Invitrogen). Primers were designed using Primer3 software (http://frodo.wi.mit.edu/primer3/input.htm) with melting temperature of 58–60 °C and production of 100–250 bp. The primers used in this study are listed in Supplementary Table S2. qRT-PCR was performed using SYBR Premix Taq Kit (TaKaRa, Dalian, China) and conducted on LightCycler 480 Detection System (Roche, Penzberg, Germany). The *PtActin* (Potri.019G010400) was used as internal control.

The expression data of *Arabidopsis NHX* genes was download from AtGenExpress database (http://jsp.weigelworld.org/expviz/expviz.jsp). *Arabidopsis* seedlings were treated under various stresses, such as heat (38 °C and recovered at 25 °C), cold (4 °C), salt (150 mM NaCl), osmotic stress (300 mM mannitol), genotoxic stress (1.5 µg/ml bleomycin +22 µg/ml mitomycin), oxidative stress (10 µM MV), wounding stress (punctured with pins), or 10 µM ABA. For drought stress, *Arabidopsis* seedlings were stressed by 15 min dry air stream (clean bench) until 10% loss of fresh weight, then incubation in closed vessels in the climate chamber.

### Integrated network analyses

The co-expression data of *PtNHXs* and *AtNHXs* was obtained from Phytozome (https://phytozome.jgi.doe.gov/pz/portal.html). For the *Populus* genome-wide co-expression network construction, transcriptome data from 24 various *P. trichocarpa* tissues including five buds (predormant bud I, predormant bud II, early dormant bud, late dormant bud, and fully open bud), three leaves (immature leaf, young leaf, and first fully expanded leaf), five stems (node, inode, ammonia treatment, nitrate treatment, and urea treatment), five roots (root tip, standard root, ammonia treatment, nitrate treatment, and urea treatment), three male catkins (early from GW9592.ZK, early from GW9840.ZE, and mid from GW9911.ZK), and three female catkins (early from BESC423.ZL, late from BESC842, and receptive from BESC442.ZG) were used to construct the genome-wide co-expression network. The *PtNHXs* co-expression networks were constructed based on the Pearson correlations (>0.85). The protein-protein interaction (PPI) data of PtNHXs was obtained from the STRING database (http://string-db.org)^31^. Cytoscape^32^ was used to visualize the resulting networks.

### Statistical analysis

The statistical analysis was performed using Duncan test and Fisher’s protected least significant difference (LSD) test of DPS (Zhejiang University, China) at 0.05 probability levels.

## Results

### Genome-wide identification of *NHX* genes in *P. trichocarpa*

To identify the *Populus NHX* genes, a tBLASTn search was performed and total of eight *NHX* genes were identified from *P. trichocarpa* genome. These genes were named according to their sequence similarity to the *NHX* genes in *Arabidopsis* and rice. All the information of the eight *PtNHX* genes, including gene locus, gene length, and deduced amino acids, was shown in Table 1. The protein length PtNHXs varied from 528 (PtNHX5) to 1,147 (PtNHX8). The molecular weights (MW) varied from 58.7 (PtNHX5) to 127 (PtNHX8) kDa; while the predicted isoelectric points (pI) were from 5.47 (PtNHX6) to 8.76 (PtNHX4) (Table 1). To validate the sequence accuracy of *PtNHX* genes from *P. trichocarpa* genome database, three *PtNHXs* (*−1*, *−6*, and *−8*) were cloned from *P. trichocarpa* cDNA and they showed high identities (99.45%, 99.57%, and 99.54%, respectively) with the sequences from *P. trichocarpa* genome database (Supplementary Fig. S1).

**Table 1:** *NHX* gene family in *P. trichocarpa*.

Group	Gene Name	Gene Locus	CDS (bp)	ORF (aa)	pI	MW (kDa)	*Arabidopsis* orthologous locus
Vac	*PtNHX1*	Potri.005G045100	1,635	544	7.30	60.4	AT3G05030
	*PtNHX2*	Potri.013G031700	1,638	545	7.67	60.2	AT3G05030
	*PtNHX3*	Potri.013G026600	1,722	573	8.03	63.5	AT5G55470
	*PtNHX4*	Potri.010G031500	1,611	536	8.76	59.6	AT3G06370
	*PtNHX5*	Potri.014G134900	1,587	528	8.68	58.7	AT3G06370
Endo	*PtNHX6*	Potri.016G000200	1,614	537	5.47	58.9	AT1G79610
PM	*PtNHX7*	Potri.008G140700	3,438	1,145	6.39	126.8	AT2G01980
	*PtNHX8*	Potri.010G100900	3,444	1,147	6.51	127.0	AT2G01980

### Phylogenetic and gene structure analyses of *PtNHX* genes

**Figure 1:**
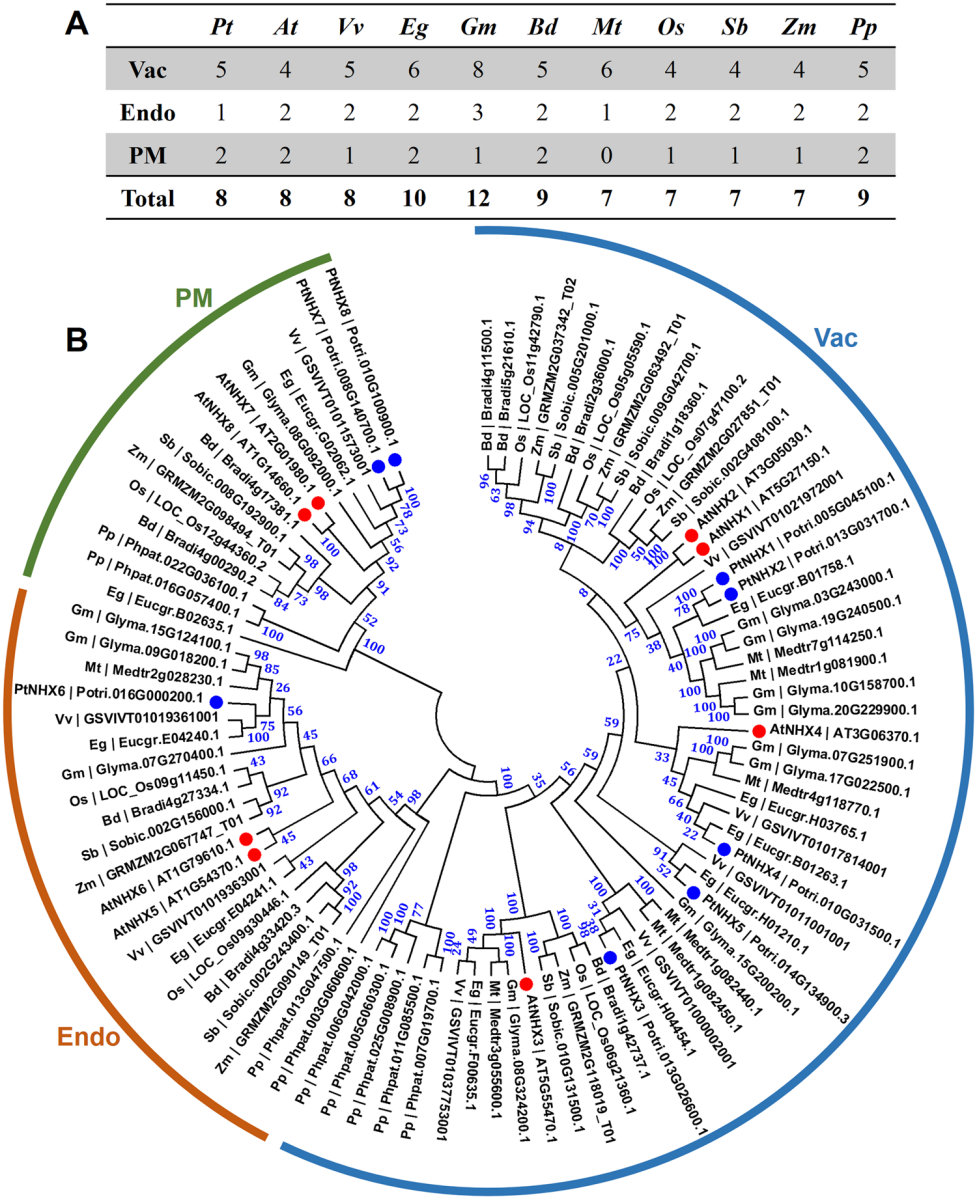
NHX members (**A**) and phylogenetic relationships (**B**) from 11 plant species. (**A**) NHX family members of *P. trichocarpa* (*Pt*), *A. thaliana* (*At*), *V. vinifera* (*Vv*), *E. grandis* (*Eg*), *G. max* (*Gm*), *M. truncatula* (*Mt*), *B. distachyon* (*Bd*), *O. sativa* (*Os*), *S. bicolor* (*Sb*), *Z. mays* (*Zm*), and *P. patens* (*Pp*). (**B**) Phylogenetic tree was constructed using the maximum likelihood (ML) method with 1,000 bootstrap replicates. The three major classes (Vac-, Endo-, and PM-) are marked with different colors. Details of *NHXs* from 11 plant species was listed in Supplementary Table S1.

To explore the evolutionary relationship of NHXs in plant kingdom, we compared PtNHXs with NHXs from other 10 species. Except for *Populus trichocarpa*, five dicotyledonous angiosperms: *Arabidopsis thaliana* (At), *Eucalyptus grandis* (Eg), *Medicago truncatula* (Mt), *Vitis vinifera* (Vv), and *Glycine max* (Gm); four monocotyledonous angiosperms: *Oryza sativa* (Os), *Sorghum bicolor* (Sb), *Brachypodium distachyon* (Bd), and *Zea mays* (Zm); and one moss: *Physcomitrella patens* (Pp) were analyzed. The size of *NHX* families in these species varied from 7 to 12 (Fig. 1A). Then, we constructed a phylogenetic tree including 92 *NHX* members from the 11 species. As shown in Fig. 1B, the *NHXs* were grouped into three subfamilies (Vac-, Endo-, and PM-classes). Vac-class *NHXs* are the most abundant *NHXs* in all the detected species. Interestingly, no PM-class *NHX* was identified in *Medicago*.

**Figure 2:**
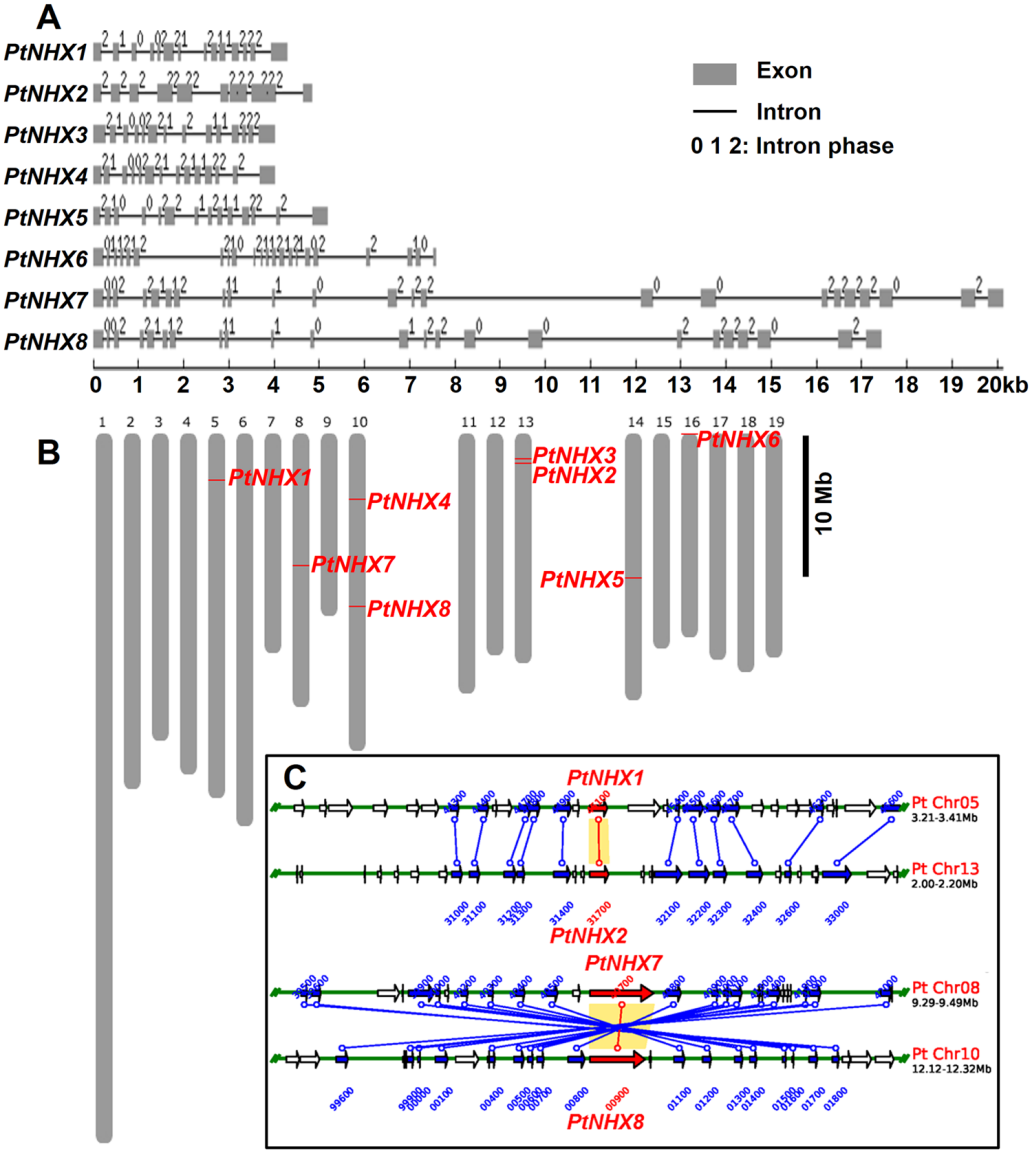
*PtNHX* gene structure, map position, and duplication analysis. (**A**) Gene structures of the *PtNHXs*. (**B**) Location of *PtNHXs* on *P. trichocarpa* chromosomes. (**C**) The duplicated *PtNHXs* (red labelled) from PGDD database.

**Figure 3:**
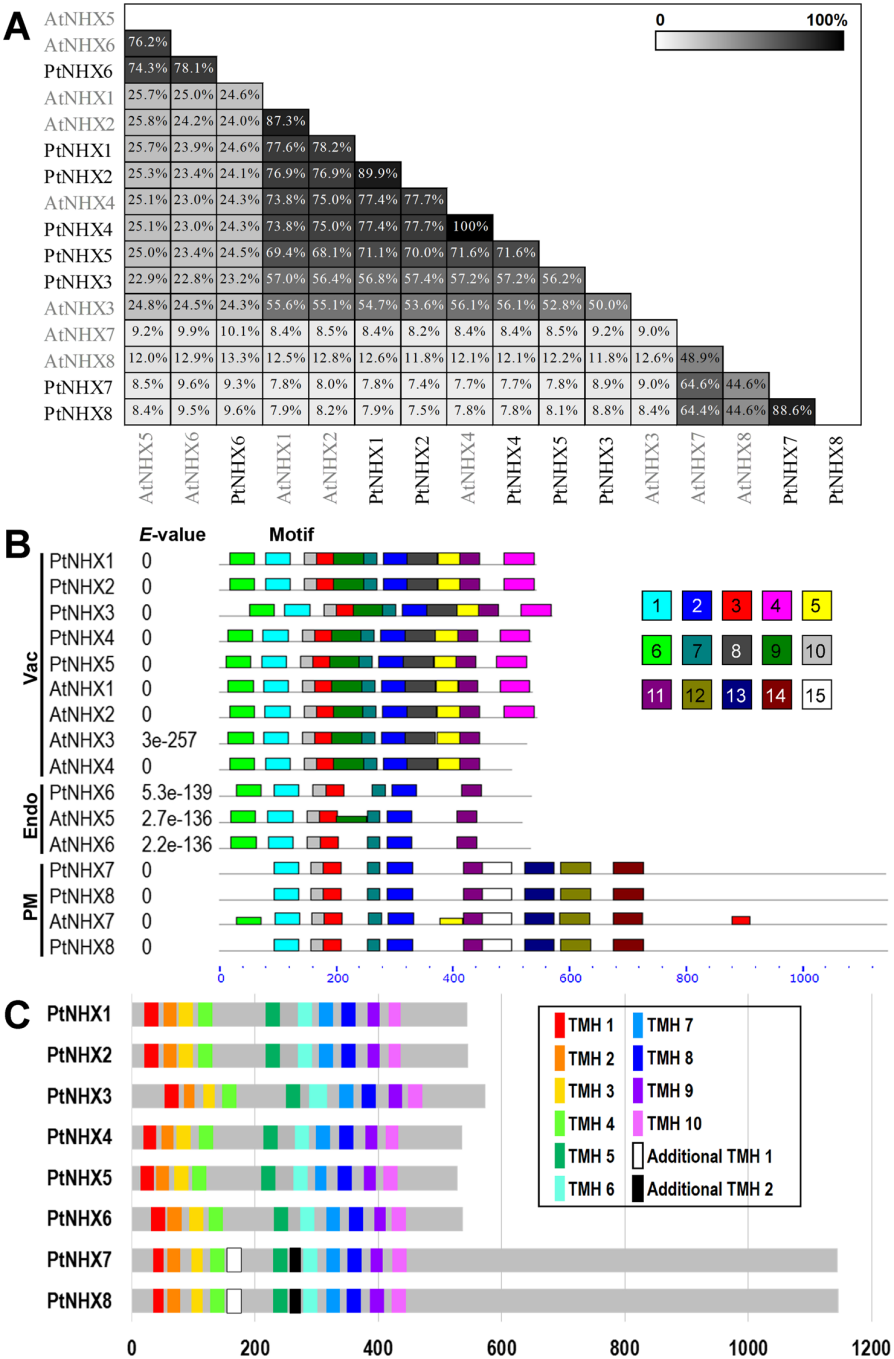
Sequence identity and conserved motifs in the NHX family members. (**A**) Pairwise sequence identity among *Populus* and *Arabidopsis* NHX proteins. (**B**) Conserved motifs of PtNHXs and AtNHXs identified by MEME. Details of different motifs indicated by different colors are shown in Fig. S4. (C) The transmembrane helices of PtNHX proteins were predicted using TMHMM2.

To analyze the structural characteristics of the *Populus NHX* genes, we compared the exon-intron organizations of these genes (Fig. 2A). Among the *PtNHX* genes, Vac-class *NHXs* (*PtNHX1–5*) had 13 introns, Endo-class *NHX* (*PtNHX6*) had 21 introns, while PM-class *NHXs* (*PtNHX7* and *PtNHX8*) had 22 introns. The intron number, exon length, and intron phase were relatively conserved among the members in the same subfamily. In addition, the sequence conservation among PtNHX proteins was also supported by amino acid sequence identity (Fig. 3A). Two PtNHX paralogous pairs exhibited high sequence identities in amino acid level (PtNHX1/PtNHX2 = 89.9% and PtNHX7/PtNHX8 = 88.6%), while the proteins in different PtNHX subfamilies showed low identity (7.4–25.8%).

### Chromosomal location and expansion analyses of *PtNHX* genes

We further analyzed the gene duplication events to understand the evolution of the *PtNHX* gene family. Eight *PtNHX* genes were mapped onto six of total 19 *P. trichocarpa* chromosomes unevenly (Fig. 2B). Two chromosomes (Chr10 and Chr13) contained two *PtNHX* genes and four chromosomes (Chr5, Chr8, Chr14, and Chr16) contained only one *PtNHX* gene on each (Fig. 2B). As a main mechanism of gene family expansion, gene duplication events provide opportunities for the new gene production and its functional divergence^33^. The paralogous genes were generated during the divergent evolution from a common ancestral gene through duplication events (segmental or tandem duplication)^34^. In poplar, only two *PtNHX* paralogous pairs (*PtNHX1*/*PtNHX2* and *PtNHX7*/*PtNHX8*) were identified and both were generated by segmental duplication events (Fig. 2C), which was similar with the duplication events of *NHXs* in *Arabidopsis* (Supplementary Fig. S2). Therefore, the expansion of *NHX* genes in poplar was mainly attributed by segmental duplications.

In *Populus*, the recent large-scale genome duplication event was occurred in 13 million years ago (MYA)^22^. The non-synonymous (*K*a) or synonymous (*K*s) substitution rates and their ratio (*K*a/*K*s) of the two *PtNHX* paralogous gene pairs showed high similarities (Table 2). The *K*a/*K*s ratio could be used to evaluate whether Darwinian selection was involved in the duplication events. The ratio >1 or <1 implying that the genes underwent a positive Darwinian selection or a purifying selection^34^. The *K*a/*K*s of the two *PtNHX* pairs were less than 0.3, indicating that purifying selection played a dominative role in *PtNHXs* duplication. According to the divergence rate (λ = 9.1 × 10^–9^) for *Populus* ^28^, the dates of the two *PtNHX* paralogous pairs were estimated in 12.09 MYA (*PtNHX1*/*PtNHX2*) and 10.99 MYA (*PtNHX7*/*PtNHX8*), indicating that the two *PtNHX* paralogous pairs were likely generated during the stage of the recent *Populus* large-scale genome duplication event (~13 MYA).

**Table 2:** Divergence between paralogous *PtNHX* gene pairs.

Gene 1	Gene 2	*K*a	*K*s	*K*a/*K*s	Date (MYA)
*PtNHX1*	*PtNHX2*	0.05	0.22	0.227	12.09
*PtNHX7*	*PtNHX8*	0.05	0.20	0.250	10.99

Notes: Synonymous (*K*s) and nonsynonymous substitution (*K*a) rates are presented for each pair.

In addition, the sliding window analysis was carried out to identify the *K*a/*K*s ratio across the full length of *PtNHX* paralogous pairs. As shown in Supplementary Fig. S3, the conserved domains of *PtNHXs* underwent strong purifying selection in both *PtNHX1/PtNHX2* and *PtNHX7/PtNHX8* paralogous pairs. While one exception region in C-terminus of *PtNHX1/PtNHX2* pair showed high *K*a/*K*s ratio (*K*a/*K*s > 1), implying that the regions corresponding to conserved domains and non-conserved domains were evolved under different selective pressure (Supplementary Fig. S3). These results indicated that purifying selection played important roles in the evolution of *NHX* gene family in *Populus*.

### Motifs characterization of PtNHX proteins

To further investigate the characteristic region of PtNHX proteins, the motif distributions in PtNHX and AtNHX proteins were analyzed using MEME and total of 15 individual motifs were identified (Fig. 3B and Supplementary Fig. S4). As predicted, the members with highly phylogenetic relationships had common motif composition. Six motifs (motif 1, 2, 3, 7, 10, and 11) were existed in all the members in the NHX family, and these motifs were enriched in the N-terminus of NHXs. Motif 6 was existed in Vac- and Endo-classes NHXs, three motifs (motif 5, 8, and 9) were only existed in Vac-class NHXs, and four motifs (motif 12, 13, 14, and 15) were only existed in PM-class NHXs. In addition, motif 4 was also Vac-class specific motif, but two AtNHXs (AtNHX3 and AtNHX4) in this subfamily were lost this motif (Fig. 3B).

Similar with other typical NHX proteins, PtNHXs containing 10 transmembrane helices (TMH1-TMH10) and a hydrophilic C-terminal domain (Fig. 3C and Supplementary Fig. S5). Despite the PM-class NHXs were significantly different from other two classes NHXs in length or sequence similarity (Fig. 3A), the 10 TMHs (TMH1-TMH10) could be detected in all of the PtNHXs. Moreover, two additional TMHs (Additional TMH1 and Additional TMH2) were identified in PM-class NHXs after TMH4 and TMH5, respectively (Fig. 3C and Supplementary Fig. S5).

### Structural analysis of PtNHX proteins

**Figure 4:**
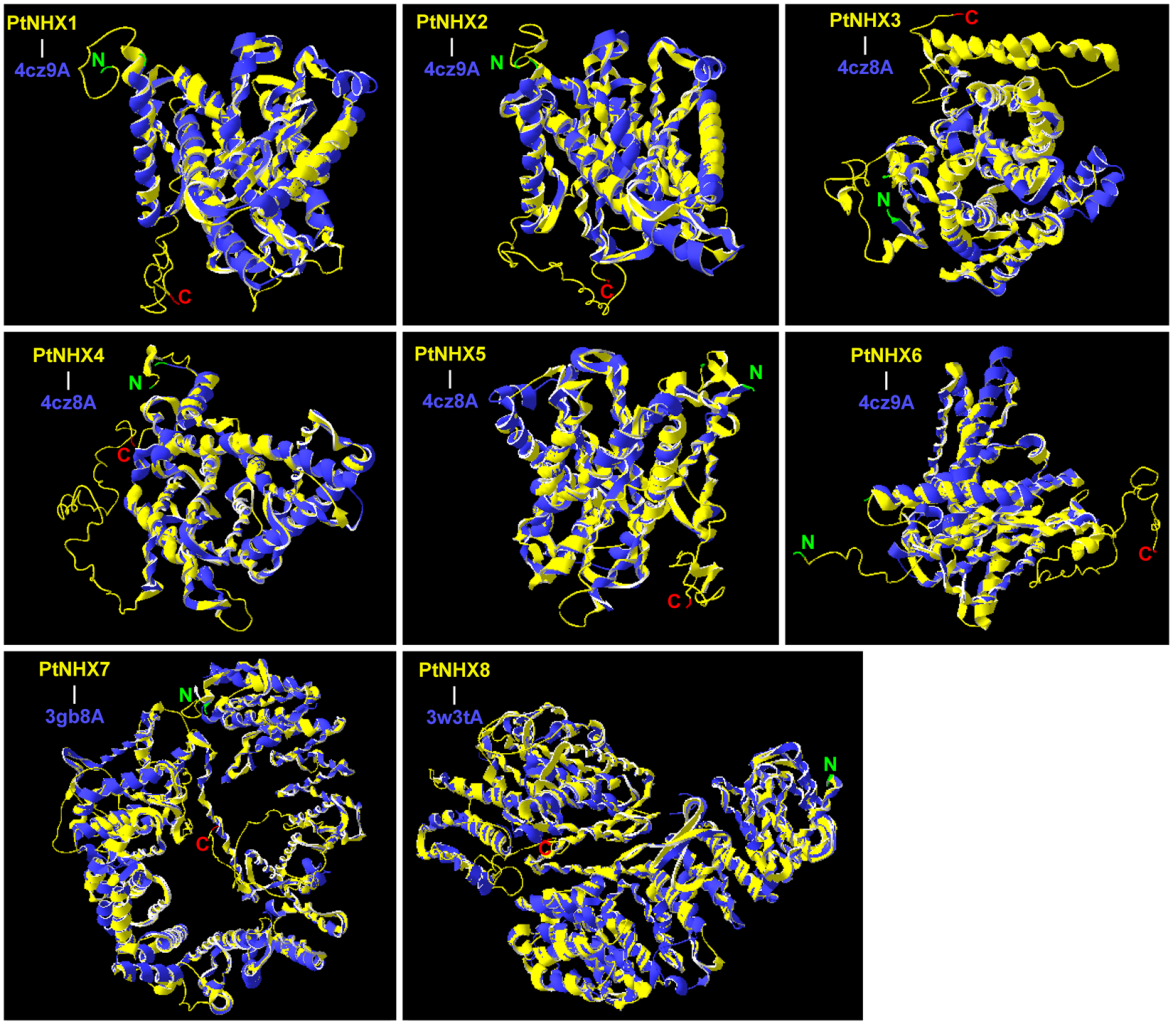
Structural analysis of PtNHX proteins. Yellow chains indicate the PtNHX proteins and blue chains indicate the best identified structural analogs in PDB for each PtNHX protein (Table 3). Details of secondary structure of PtNHX proteins are shown in Fig. S5.

In order to understand the functional mechanism of PtNHX proteins, we analyzed the 3D structures of PtNHXs. According to the best structural templates and crystal structures from Protein Data Bank (PDB), we constructed the best predicted model for the eight PtNHX proteins. To quantify the confidence of constructed model, C-score was used to evaluate the predicted protein model. Generally, the C-score range from −5 to 2, higher value means the model with higher confidence. In our study, the predicted PtNHX models were high accuracy with C-score ranged from −1.6 to 0.08 (Table 3), indicating that the protein structures were constructed with high accuracy. As shown in Fig. 4 and Supplementary Fig. S6, the TMHs compose a hollow cylinder and embedded in the membrane to provide the channel for Na^+^ and H^+^ transport.

**Table 3:** Structural dependent modeling parameters for the PtNHX proteins.

	C-score	TM-score	RMSD (Å)	Best identified structural analogs in PDB				
				PDB Hit	TM-score^a^	RMSD^a^	IDEN^a^	Cov
PtNHX1	−1.25	0.56 ± 0.15	10.4 ± 4.6	4cz9A	0.711	1.16	0.213	0.724
PtNHX2	−1.15	0.57 ± 0.15	10.2 ± 4.6	4cz9A	0.706	1.25	0.216	0.721
PtNHX3	−0.77	0.62 ± 0.14	9.4 ± 4.6	4cz8A	0.668	1.34	0.217	0.682
PtNHX4	0.08	0.72 ± 0.11	7.3 ± 4.2	4cz8A	0.722	0.97	0.204	0.731
PtNHX5	−1.6	0.52 ± 0.15	11.2 ± 4.6	4cz8A	0.723	1.48	0.199	0.742
PtNHX6	−0.37	0.67 ± 0.13	8.3 ± 4.5	4cz9A	0.714	1.13	0.203	0.726
PtNHX7	−1.46	0.53 ± 0.15	12.9 ± 4.2	3gb8A	0.771	1.04	0.097	0.777
PtNHX8	−0.9	0.60 ± 0.14	11.4 ± 4.5	3w3tA	0.851	1.28	0.086	0.859

C-score [−5, 2] is the confidence of each model, higher value signifies a model with a higher confidence and vice-versa. TM-score and RMSD are estimated based on C-score and protein length following the correlation observed between these qualities. TM-score^a^ is TM-score of the structural alignment between the query structure and known structures in the PDB library. RMSD^a^ is the RMSD between residues that are structurally aligned by TM-align. IDEN^a^ is the percentage sequence identity in the structurally aligned region. Cov represents the coverage of the alignment by TM-align and is equal to the number of structurally aligned residues divided by length of the query protein.

### Various *cis*-acting elements in the *PtNHX* promoters

**Figure 5:**
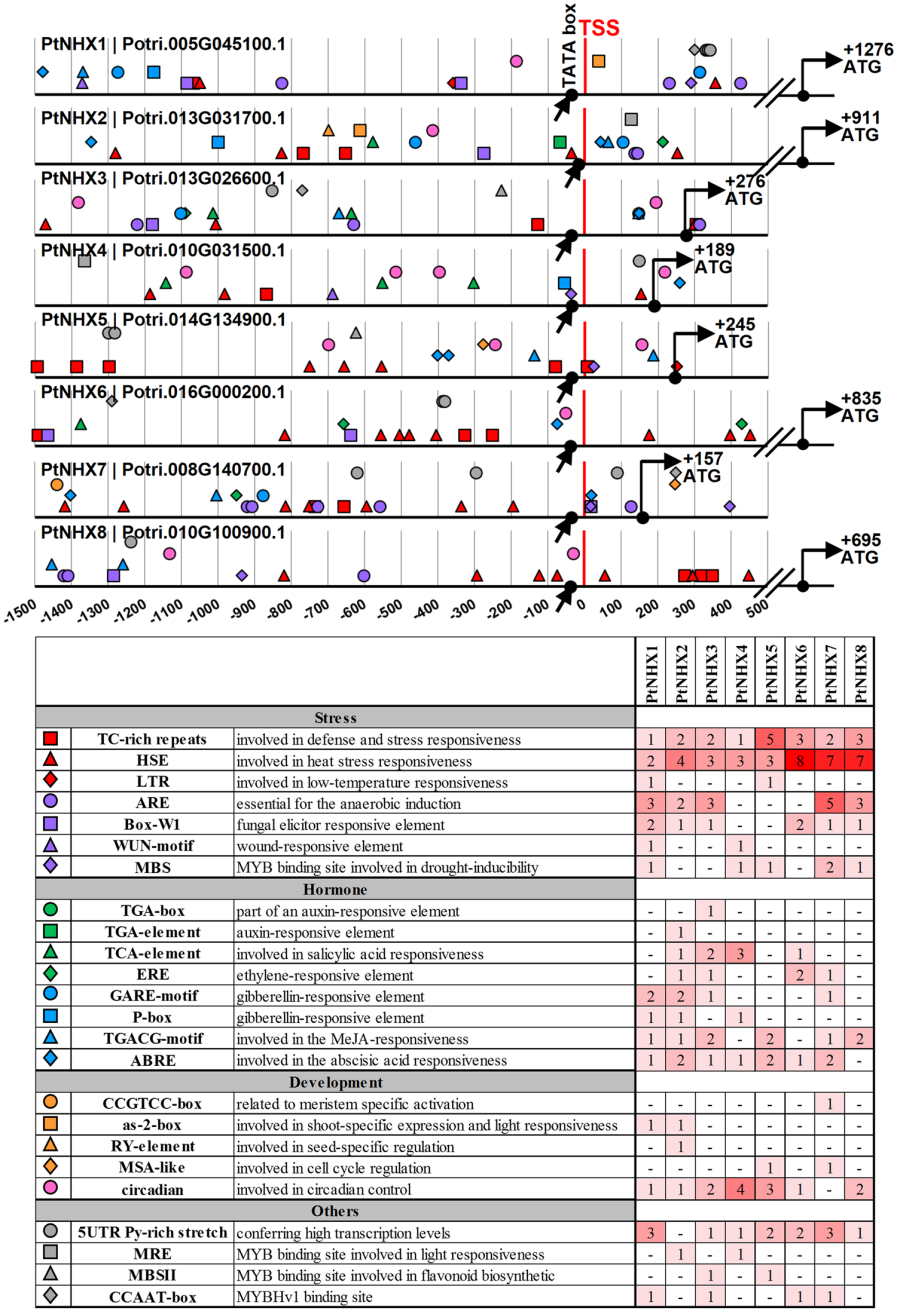
*cis*-acting elements in *PtNHX* promoters. Schematic representation of promoter regions of eight *PtNHXs*. Distances are relative to the transcription start site (TSS).

To further understand the potential transcriptional regulatory mechanism of transcription factors to *PtNHX* genes, the sequences of −1,500 bp to +500 bp relative to the transcription start sites (TSS) of the eight *PtNHXs* were used to identify the *cis*-acting elements. As shown in Fig. 5, amount of stress-related (e.g. heat, low temperature, drought, wound, and defense) and hormone-related (e.g. auxin, ethylene, GA, SA, MeJA, and ABA) elements were identified in the *PtNHX* promoters. Among the stress-related *cis*-acting elements, total of 19 TC-rich repeats and 37 HSE were identified in the eight *PtNHX* promoters. Among the hormone-related *cis*-acting elements, the ABRE (*cis*-elements involved in ABA responsiveness) were found in seven of eight *PtNHX* promoters. Noticeably, the HSE were enriched in the promoter regions of *PtNHX6*, *PtNHX7*, and *PtNHX8* (with eight, seven, and seven HSEs, respectively). The results suggested that *PtNHXs* might have potential roles in stress adaptation and various hormone signal responsiveness.

### Expression patterns of *PtNHXs* across different tissues and response to stresses

**Figure 6:**
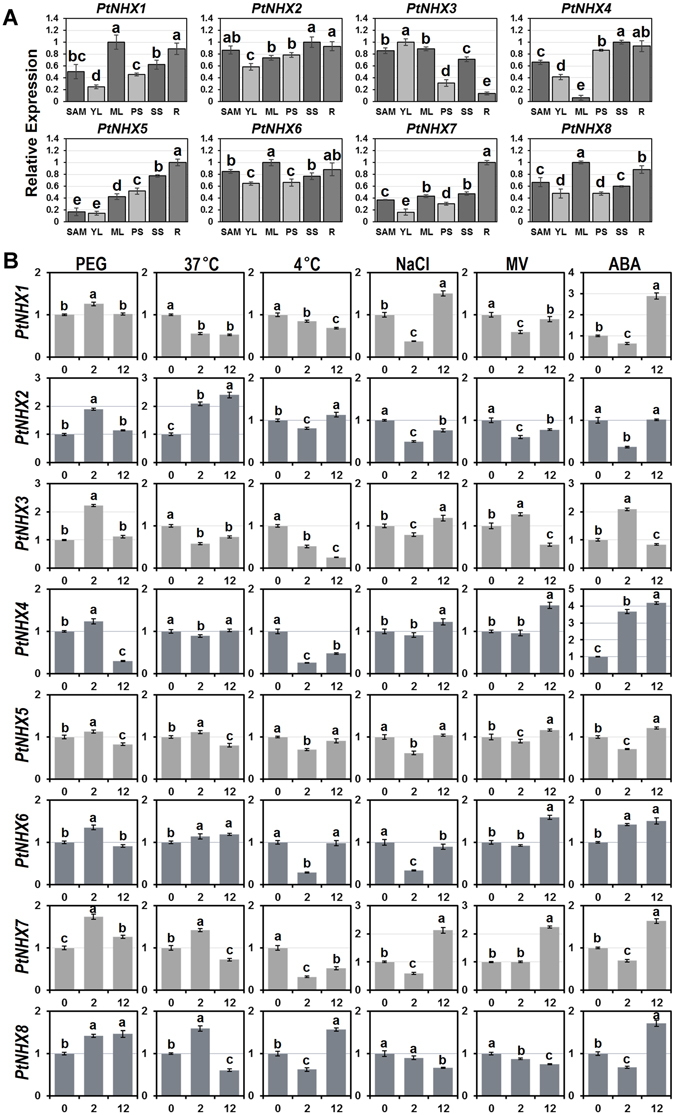
Expression patterns of *PtNHX* genes in different tissues (**A**) and under various abiotic stresses (**B**) using qRT-PCR. (**A**) The relative mRNA abundance of the *PtNHXs* were quantified in six tissues (SAM–shoot apical meristems, YL–young leaves, ML–mature leaves, PS–primary stem, SS–secondary stem, R–roots). (**B**) The expression patterns of the *PtNHXs* at 2 and 12 hours after treated with drought (10% PEG), heat (37 °C), cold (4 °C), salt (150 mM NaCl), oxidative stress (100 μM MV) or 100 μM ABA. Bars with the same letters are not significantly (*P* < 0.05).

To investigate the potential roles of *PtNHXs* in growth and/or development, their expression patterns across six poplar tissues were analyzed using qRT-PCR. Six tissues including shoot apical meristem (SAM), young leaves (YL), mature leaves (ML), primary stems (PS), secondary stems (SS), and roots (R) were selected for study. As shown in Fig. 6A, four genes (*PtNHX1*, *PtNHX3*, *PtNHX6*, and *PtNHX8*) were highly expressed in mature leaf, while two (*PtNHX5* and *PtNHX7*) were highly expressed in root. *PtNHX4* was highly expressed in both stem (including primary stem and secondary stem) and root; similarly, its homologous gene *AtNHX4* was highly expressed in root and stem (Supplementary Fig. S7). We also compared the expression patterns between *PtNHXs* in each paralogous pair, the expression patterns of paralogous *PtNHXs* were significant different in both *PtNHX1/PtNHX2* pair and *PtNHX7/PtNHX8* pair, indicating that their expression were divergent during the evolution though the paralogous genes were duplicated from the same original gene.

Moreover, the expression patterns of eight *PtNHXs* under drought, heat, cold, salinity, oxidative stress, or ABA treatments were analyzed (Fig. 6B). Because all the eight *PtNHXs* could be detected in leaves, we selected the first fully expanded leaves to confirm their responses to various abiotic stresses or hormone signal. Under drought stress, all of *PtNHX* genes were up-regulated at 2 h after PEG treatment. Similarly, the expression of most *AtNHXs* were induced in *Arabidopsis* seedlings under drought and osmotic stresses (Supplementary Fig. S8). *PtNHX2* was immediately up-regulated under heat stress, its expression pattern was consistent with its orthologous in *Arabidopsis* (*AtNHX2*, Supplementary Fig. S8). When the poplar seedlings were exposed to salinity stress, two *PtNHXs* (*PtNHX1* and *PtNHX7*) were dramatically induced after 12 h treatment. The orthologous of *PtNHX7* was well known as the salt tolerance locus *SOS1* in *Arabidopsis*, which plays crucial role in root Na^+^ long distance transport to shoot^20^. As an important plant hormone, ABA is involved in not only plant development but also response to various stresses^35^. As shown in Fig. 6B, the expression of all of the eight *PtNHXs* were affected by ABA treatment, implying that the members in *PtNHX* family might be involved in ABA-dependent signal pathway. All the expression data indicated that most of the *PtNHXs* were responsive to certain abiotic stresses at the transcriptional level. Similar with that in poplar, the members of *NHX* family in *Arabidopsis* were also response to various abiotic stresses (Supplementary Fig. S8). *NHX* genes might be commonly involved in plant adapt to various environmental stress conditions in different species.

### Co-expression network of *PtNHX* genes

**Figure 7:**
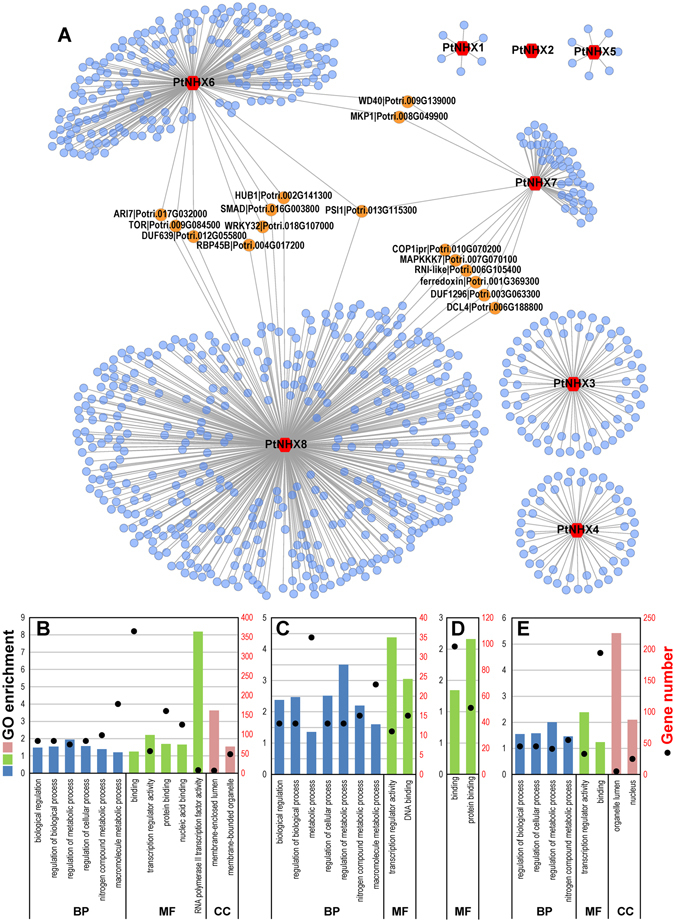
Co-expression network of *PtNHX* gene family. (**A**) *PtNHXs* are represented as red hexagon, hub co-expressed genes are represented as orange nodes. Partial results of GO enrichment and enriched gene number of all the genes co-expressed with the eight *PtNHXs* (**B**), *PtNHX3* (**C**), *PtNHX6* (**D**), and *PtNHX8* (**E**) were shown. Details of GO enrichment are listed in Fig. S9. BP, biological process; MF, molecular function; CC, cellular component.

To explore the potential molecular functions of *PtNHXs*, the co-expression network of *PtNHXs* was constructed based on the genome-wide gene expression patterns. As shown in Fig. 7, total of 6, 68, 51, and 7 genes were co-expressed with *PtNHX1*, *PtNHX3*, *PtNHX4*, and *PtNHX5*, respectively; and the genes co-expressed with the four *PtNHXs* are independent. No gene was co-expressed with *PtNHX2*. Different with those *PtNHXs* co-expressed with independent genes, three *PtNHXs* (*PtNHX6*, *PtNHX7*, and *PtNHX8*) with more co-expressed genes (199, 55, and 422, respectively) and they share several hub co-expressed genes. Among these hub genes, PIS (Potri.013G115300) was co-expressed with all the three *PtNHXs*, which is located in plasma membrane and required for growth (Table 4). Two salt stress related genes MKP1 (Potri.008G049900) and WD40 (Potri.009G139000) were co-expressed with both *PtNHX6* and *PtNHX7*. In addition, seven genes were co-expressed with *PtNHX6* and *PtNHX8*, six genes were co-expressed with *PtNHX7* and *PtNHX8* (Fig. 7A and Table 4).

**Table 4:** Annotation of hub genes in the *PtNHX* co-expression network.

Gene Name	Gene ID	*Arabidopsis* orthologous	Functional Description	Co-expressed with *PtNHXs*
PSI1	Potri.013G115300	AT1G34320	Located in plasma membrane, required for growth.	*PtNHX6*, *PtNHX7*, and *PtNHX8*
MKP1	Potri.008G049900	AT3G55270	Involved in protein dephosphorylation, inactivation of MAPK activity, response to salt stress, UV, and fungus.	*PtNHX6* and *PtNHX7*
WD40	Potri.009G139000	AT4G35140	Regulated by NAC2 in salt stress response and lateral root development.	*PtNHX6* and *PtNHX7*
HUB1	Potri.002G141300	AT2G44950	E3 uniquitin ligases involved in monoubiquitination of histone H2B. Involved in cell devision, cell growth and defense response.	*PtNHX6* and *PtNHX8*
SMAD	Potri.016G003800	AT5G47790	SMAD/FHA domain-containing protein	*PtNHX6* and *PtNHX8*
WRKY32	Potri.018G107000	AT4G30935	Regulation of transcription.	*PtNHX6* and *PtNHX8*
RBP45B	Potri.004G017200	AT1G11650	RNA binding protein involved in mRNA processing, response to cytokinin and ozone.	*PtNHX6* and *PtNHX8*
TOR	Potri.009G084500	AT1G50030	Regulate cell growth in response to nutrient availability.	*PtNHX6* and *PtNHX8*
DUF639	Potri.012G055800	AT1G48840	Plant protein of unknown function (DUF639)	*PtNHX6* and *PtNHX8*
ARI7	Potri.017G032000	AT2G31510	Positive regulation of proteasomal ubiquitin-dependent protein catabolic process.	*PtNHX6* and *PtNHX8*
DCL4	Potri.006G188800	AT5G20320	Rnase III-like enzyme catalyzes processing of trans-acting small interfering RNA precursors in a distinct small RNA biogenesis pathway.	*PtNHX7* and *PtNHX8*
COP1ipr	Potri.010G070200	AT5G43310	Located in plasma membrane, positive regulator of light-regulated genes.	*PtNHX7* and *PtNHX8*
ferredoxin	Potri.001G369300	AT4G26620	Sucrase/ferredoxin-like family protein	*PtNHX7* and *PtNHX8*
MAPKKK7	Potri.007G070100	AT3G13530	Involved in cell cycle, cell division, plasma membrane organization, pollen development, regulation of cell division, regulation of embryonic development, regulation of extent of cell growth, regulation of unidimensional cell growth.	*PtNHX7* and *PtNHX8*
RNI-like	Potri.006G105400	AT5G01720	Involved in ubiquitin-dependent protein catabolic process.	*PtNHX7* and *PtNHX8*
DUF1296	Potri.003G063300	AT3G13990	Kinase-related protein of unknown function (DUF1296)	*PtNHX7* and *PtNHX8*

We then performed the GO enrichment analysis of the co-expressed genes to reveal their potential function. Among the genes co-expressed with the eight *PtNHXs*, 29 biological process (BP), 11 molecular function (MF), and 11 cellular component (CC) terms were significant enriched. For the BP, the enriched terms are related with “regulation of biological process”, “regulation of metabolic process”, “regulation of cellular process”, “nitrogen compound metabolic process”, and “macromolecule metabolic process”. The enriched MF terms are related with “binding”, “transcription regulation activity”, and “RNA polymerase II transcription factor activity”. And the enriched CC terms are related with “membrane-enclosed lumen” and “membrane-bounded organelle” (Fig. 7B and Supplementary Fig. S9). Separately GO enrichment analysis of co-expressed genes for each *PtNHXs* indicated only the genes co-expressed with three *PtNHXs* (*PtNHX3*, *PtNHX6*, and *PtNHX8*) enriched in specific GO terms. For *PtNHX3*, the co-expressed genes enriched in the BP terms “biological regulation” and “metabolic process” and the MF terms “transcription regulator activity”. The genes co-expressed with *PtNHX6* were enriched in the MF terms “binding” and specifically in “protein binding”. For *PtNHX8*, the co-expressed genes were enriched in the BP term “regulation process”, the MF term “transcription regulator activity”, and the CC terms “organelle lumen” and “nucleus”.

To reveal the evolutionary divergence of *NHX* genes in model woody species *Populus* and model plant *Arabidopsis*, we then constructed the co-expression network of *Arabidopsis NHX* genes and compared the differences between *PtNHX* co-expression network and *AtNHX* co-expression network. Different with the co-expression pattern of *PtNHXs*, three *AtNHXs* (*AtNHX4*, *AtNHX6*, and *AtNHX7*) were connected by a series co-expressed genes in *Arabidopsis NHX* co-expression network (Supplementary Fig. S10). The co-expressed genes indicated that strong co-expression relationship between *AtNHX6* and *AtNHX7* while weak co-expression relationship between *AtNHX4* and *AtNHX6*. To make the two co-expression networks (*PtNHXs* and *AtNHXs* co-expression networks) comparable, the GO enrichment were performed based on the genes co-expressed with each *NHX* gene in the two network. The GO enrichment analysis of *AtNHX* genes showed that the genes co-expressed with *AtNHX4* were enriched in the BP terms “developmental process”, “cellular component organization” and “cellular signal pathway”; the genes co-expressed with *AtNHX6* were enriched in the BP terms “biological regulation”, “developmental process”, and “reproduction”; while the genes co-expressed with *AtNHX7* were enriched in the BP terms “biological regulation”, “developmental process”, “localization”, and “reproduction” (Supplementary Table S3). The co-expressed networks of *PtNHXs* and *AtNHXs* indicated that the *NHX* genes might be function in developmental processes and/or stress responses through cooperate with other functional genes.

### Protein-protein interaction (PPI) network of PtNHXs

**Figure 8:**
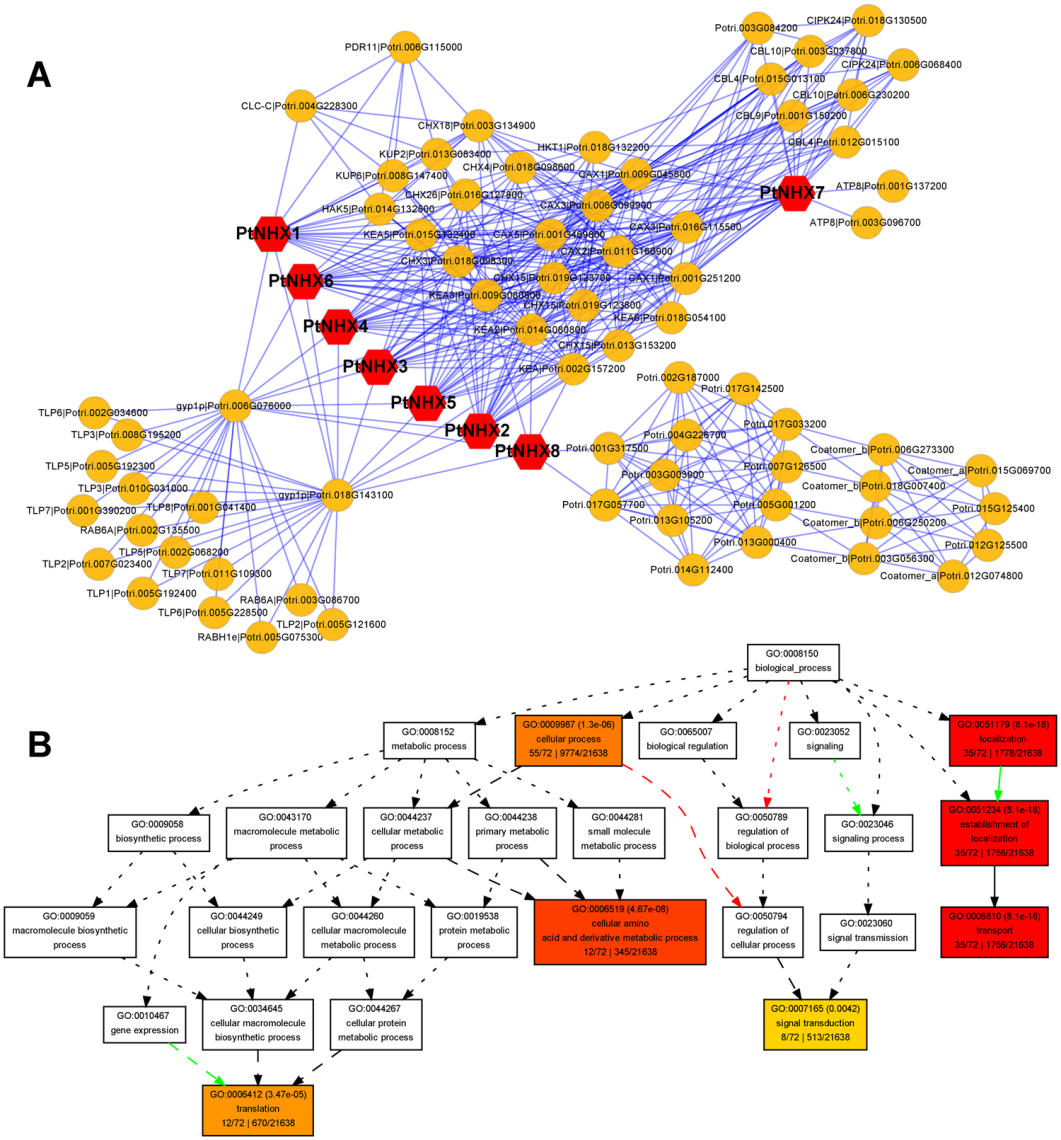
Protein-protein interaction (PPI) network of PtNHXs. (**A**) PPI network of PtNHX proteins. (**B**) Enriched BP terms of genes coding the proteins in the PtNHX PPI network. Complement GO enrichment data is shown Fig. S11.

To further reveal the function of PtNHXs during the interaction with other proteins, we constructed a PPI network. As shown in Fig. 8A, all the eight PtNHXs share most of the same interacted proteins. In addition, some of proteins were specifically interact with PtNHX7 and PtNHX8 separately. The proteins interacted with all the PtNHXs including CHX, CAX, KEA, HKT, and KUP proteins. Two CIPK and four CBL proteins specific interacted with PtNHX7, while six coatomer proteins specific interacted with PtNHX8. To detect the functional abundance of proteins interacted with PtNHXs, the GO enrichment analysis was performed based on the PPI network. The enriched BP terms of proteins interacted with PtNHXs including “cellular process”, “transport”, and “signal transduction” (Fig. 8B and Supplementary Fig. S11).

### Natural variation in *PtNHX* genes

**Figure 9:**
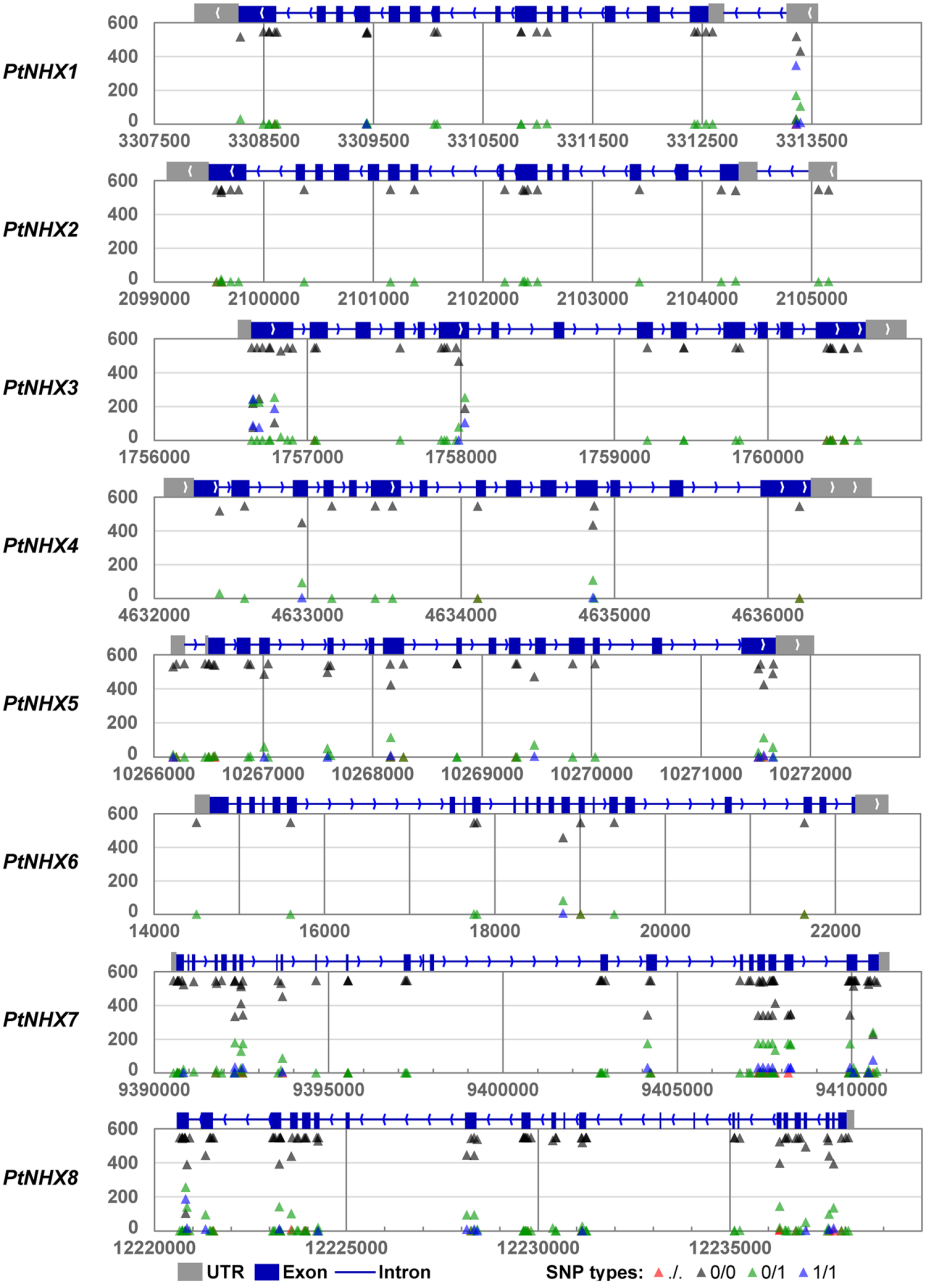
Identification of SNPs in *PtNHX* gene family from 549 *P. trichocarpa* individuals.

Generally, the variation located in gene body might generate functional divergence or associate some traits. Based on the whole genome re-sequence data of 549 *P. trichocarpa* natural individuals in North America, we identified the single nucleotide polymorphisms (SNPs) in *PtNHX* genes. The SNPs located in gene region could be classified into different types according to their consequence, such as synonymous, non-synonymous, start gained, start lost, codon deletion, frame shift, splice site acceptor, splice site donor, and stop gained. Here we omit the synonymous SNPs because they will not affect the composition of their coding proteins. A total of 274 SNPs were detected in the eight *PtNHX* genes, among the detected SNPs 249 were non-synonymous coding SNPs (Table 5). The number of SNPs was significant different in the *PtNHX* genes, total of 77 SNPs were detected in *PtNHX7* while only 8 SNPs were detected in *PtNHX6*. As membrane located proteins, the SNPs in the TMH regions might play important roles in their location. No SNP was located in the TMH regions of *PtNHX6*, while 15 and 17 SNPs were located in the TMH regions of *PtNHX7* and *PtNHX8*. We then analyzed the SNP type frequency of each SNP based on the *P. trichocarpa* population. The frequency of SNPs of *PtNHX7* was significantly higher than other *PtNHX* genes, especially in the C-terminal region of *PtNHX7* (Fig. 9).

**Table 5:** Summary of SNPs in *PtNHX* genes.

*PtNHXs*		*PtNHX1*	*PtNHX2*	*PtNHX3*	*PtNHX4*	*PtNHX5*	*PtNHX6*	*PtNHX7*	*PtNHX8*	Total
Gene length		5680	6106	4351	4603	5865	8135	20568	17616	
Number of SNPs^1^		22	19	33	10	30	8	77	75	274
Different type of SNPs	non-synonymous coding	16	17	32	10	24	6	71	73	249
	codon deletion						1			1
	frame shift							2		2
	start gained	4	2			3	1	1	1	12
	start lost			1		1				2
	stop gained	1						1		2
	splice site acceptor					1		1		2
	splice site donor	1				1		1	1	4
SNPs located in TMH regions		5	2	4	3	11	0	15	17	57
TMH SNPs/Total SNPs (%)		22.7	10.5	12.1	30.0	36.7	0	19.5	22.7	22.8
Total SNPs/Gene length (%)		3.87	3.11	7.58	2.17	5.12	0.98	3.74	4.26	

^1^Only the SNPs affect the composition of the proteins were listed in here, i.e. synonymous SNPs were not analyzed in this study.

## Discussion

Ion transports play crucial roles in many aspects of biological processes, such as ions uptake or sequestration, provide energy, and cell expansion^36^. In plants, Na^+^/H^+^ antiporters (NHXs) as important members in transporters, mediate the coupled exchange of Na^+^ or K^+^ for H^+^ in all cellular compartments^36, 37^. Based on their subcellular localizations, NHXs are grouped into three classes (Vac-, Endo-, and PM-class). The members in each class of NHX family were highly similar from algae to higher plants, indicating that NHXs retained conserved functions during the evolution^38^.

In this study, total of eight *PtNHXs* were identified from woody model species *P. trichocarpa*. In contrast with *Arabidopsis*, one more Vac-class *NHX* but one less Endo-class *NHX* were identified in poplar. According to comparative genomics studies, the genes in poplar are ~1.4–1.6 fold of *Arabidopsis* homologs^22^. But the *NHX* family did not expanded as other gene families in poplar. Overall, the size of *NHX* family was similar in all the other 10 species analyzed in this study, implying that the *NHX* family might be relatively conserved during the evolution. Gene duplication is the primary mechanism of new gene production, it plays important role in gene family expansion. In *PtNHX* family, two paralogous pairs were generated by segmental duplication events in 10.99 and 12.09 MYA. This stages were consist with the ~13 MYA the recent large-scale genome duplication event in *Populus* ^22^.

It has been reported that the membrane-spanning pore and cation-binding domain were highly conserved in plants^5, 39^, which comprises of the nonapeptide “FFIYLLPPI”^40, 41^. This nonapeptide was located in the N-terminus of motif 1 and it was existed in all the detected NHXs in *Arabidopsis* and poplar (Fig. 3B and Supplementary Fig. S4). In contrast with the conserved N-terminus, the C-terminus of NHX proteins were highly diverged even in the same class NHX. In *Arabidopsis*, the N-terminus of AtNHX1 is located in the cytosol while the hydrophilic C-terminus in the vacuolar lumen. The mutation deleted the C-terminal hydrophilic region resulted in an enhanced Na^+^/H^+^ transport activity, suggesting that the C-terminus play crucial roles in not only subcellular localization but also regulation of transport activity^6, 7^. The heterogeneous C-terminus of PtNHXs provide opportunity for their functional divergence. In addition, the members in PM-class had two additional TMHs after TMH4 and TMH5, where region was also considered critical for transport activity^7^.

Nowadays, amounts of studies have indicated that *NHXs* play important roles in plant growth and development. For instance, the *Arabidopsis nhx1 nhx2* double mutant showed severe reduction in vegetative growth due to the substantial decreased in cell size^17^. In this study, *PtNHX* genes were expressed in tissue-specific patterns and were induced by various stresses, indicating that the members in *PtNHX* family might be also involved in developmental processes or stress responses in different tissues. Duplicated *PtNHXs* showed different expression patterns in various tissues (Fig. 6A), indicating that the duplication events in *PtNHX* family provide opportunities to break the functional constraint from the original gene during the evolution. In *Arabidopsis*, GUS driven by the promoter of *AtNHX7* was mainly expressed in epidermal cells of the root tips and parenchyma cells at the xylem/symplast boundary^20^. Similarly, its orthologous in poplar, *PtNHX7*, was also highly expressed in root. In addition, *PtNHX4* was highly expressed in stem and root, which was consist with the expression of its orthologous in *Arabidopsis* (*AtNHX4*, Supplementary Fig. S8). The similar expression patterns of *NHX* ortholog in poplar and *Arabidopsis* indicating that the *NHX* genes might be retained the conserved function in different species.

Stress response analyses showed that each *PtNHX* was response to at least one abiotic stress of drought, heat, cold, salinity, oxidative stress, or ABA. Noticeably, *PtNHX7* and *PtNHX8* showed significant expression changes under all the abiotic stresses. As the binding sites of transcription factors, *cis*-acting elements play important role to determine genes’ expression patterns^42^. Compared with other *PtNHXs*, promoters of *PtNHX7* and *PtNHX8* harbored more stress-related *cis*-acting elements, including HSE (involved in heat stress responsiveness, seven in both *PtNHX7* and *PtNHX8*), ARE (essential for the anaerobic induction, five in *PtNHX7* and three in *PtNHX8*), TC-rich repeats (defense and stress response, two in *PtNHX7* and three in *PtNHX8*), MBS (MYB binding site involved in drought-inducibility, two in *PtNHX7* and one in *PtNHX8*), and Box-W1 (fungal elicitor responsive element, one in both *PtNHX7* and *PtNHX8*), which might be the reason of *PtNHX7* and *PtNHX8* significant response to these stresses. Except for the stress-related *cis*-acting elements, amounts of hormone-related elements were also identified in the promoters of *PtNHXs*. In plants, various abiotic stresses could elicit the accumulation of ABA, then trigger a series of physiological and molecular responses to acclimate the environments. Seven *PtNHX* (*PtNHX1–7*) promoters containing one or two ABREs (ABA responsive *cis*-acting element), suggest *PtNHX1–7* might be involved in ABA signal pathway. Although no ABRE was detected in the promoter of *PtNHX8*, it was also induced at 12 h under ABA treatment, suggesting that there are other regulatory mechanisms in ABA responsiveness of *PtNHX8*.

As the membrane proteins, NHXs might be cooperated with other proteins. The co-expression analysis provide systematic information on gene-to-gene association^43^. Based on the co-expression network, many stress-related and transport-related genes were co-expressed with *PtNHX5*. In *Arabidopsis*, its orthologous *AtNHX4* (*At3g06370*) was localized in vacuole, and the *nhx4* mutant showed enhanced tolerance to salt stress^44^. Vacuoles segment many cellular components (e.g. ions, sugars, proteins, and secondary metabolites) and play critical roles in plant stress responses^45^. The transport-related genes co-expressed with *PtNHX5* suggest that *PtNHX5* might be involved in the vacuolar trafficking processes in poplar. Compared to the other class *PtNHXs*, the PM-class *PtNHX7* and *PtNHX8* have a long C-terminal cytosolic tail (Fig. 3), which provide more possibility to interact with other proteins^20^. Based on the PtNHX PPI network, PtNHX7 and PtNHX8 were interacted with many other proteins different with the proteins interacted with PtNHX1–6 (Fig. 8). This was consistent with the structural characteristic of long C-terminal cytosolic tails of PtNHX7 and PtNHX8. Although PtNHX7 and PtNHX8 belong to paralogous pairs generated by WGD (Fig. 2 and Supplementary Fig. S3), the protein 3D structures of PtNHX7 and PtNHX8 were significant different (Fig. 4). The structural difference between PtNHX7 and PtNHX8 provide the physical foundation for they interact with different set of proteins (Fig. 8).

As one class of calcium sensors, the calcineurin B-like (CBL) proteins can interact with and regulate the CBL-interacting protein kinases (CIPK) to mediate the calcium signal transduction. During environmental adaptation reactions, the interaction between CBL and CIPKs provide flexible and specific signal response mechanism^36, 46^. It has been proved that NHX7 (SOS1) was regulated by CBL and CIPK mediated Ca^2+^ signaling pathway during salinity response. The kinase CIPK24 was recruitment to the membrane through physical interaction with CBL4 and then active the Ca^2+^-dependent NHX7^47^. Noticeable, *CBL4* is only expressed in root^48^. Similar with the expression pattern of *CBL4*, the *PtNHX7* was highly expressed in root (Fig. 6A), the co-expression pattern of *PtNHX7* and *CBL4* in root provide opportunity of their functional interaction. The PPI network of PtNHXs indicated that PtNHX7 physically interact with CBL and CIPK proteins different with other PtNHX proteins. Recently, a meta-analysis indicates overexpression *CPA1* (or *SOS1*), the ortholog of *PtNHX7*, had statistically significant impacts for 10 of the 19 plant characteristics examined, by 25% or more. Compared to transfer into or from other genera, the *CPA1* transformed to or from *Arabidopsis* have led to smaller *CPA1*-induced increases. Heterogeneous expression of *CPA1* led to greater increases in leaf chlorophyll and root length than homologous expression^49^. This is consistent with we identified *PtNHX7* as the primary *NHX* involved in salt stress response. Our findings indicated that the members of *PtNHX* family play distinct roles in developmental processes and stress responses, and provided a comprehensive understanding of the function of *PtNHXs* in poplar.

## Conclusions

In this study, eight *NHXs* from three subfamilies (Vac-, Endo-, and PM-classes) were identified in the *P. trichocarpa* genome. Comprehensive analysis including phylogeny, gene structures, duplications, conserved motifs, tissues-specific expression and stress responses, and integrated networks (co-expression network and protein-protein interaction network) were performed. In particular, we found that the whole genome duplication (WGD) event played a dominative role in expansion of *NHX* family in poplar, and purifying selection was the main force. Protein structural analysis indicated that the THMs compose a hollow cylinder and embedded in the membrane to provide the channel for Na^+^ and H^+^ transport. In addition, the tissue-specific expression patterns of *PtNHXs* provided functional information in poplar’s development processes. Moreover, responses of the *PtNHXs* to drought, heat, cold, salinity, oxidative stress, or ABA treatment indicated that the *PtNHXs* involved in single or multiple stress responses in poplar. Co-expression network of *PtNHXs* indicated that several hub genes were connected with the End- and PM-classes *PtNHXs*. Among the eight PtNHXs, only PtNHX7 interact with CBL and CIPK suggests that PtNHX7 might be the primary NHX involved in CBL-CIPK pathway during salt stress responses. Our results contribute valuable information for future functional investigations of *PtNHX* gene family.

## Electronic supplementary material

Dataset 1

Dataset 2

Dataset 3

Supplementary data
