# Supplementary material for: Expression and integrated network analyses revealed functional divergence of NHX-type Na+/H+ exchanger genes in poplar
Source: Sci Rep. 2017 Jun 1;7:2607. doi: 10.1038/s41598-017-02894-8 (PMC5453932; doi:10.1038/s41598-017-02894-8)
Supplement: Supplementary file 4 — Supplementary data [file 41598_2017_2894_MOESM4_ESM.pdf]

Supplementary Information

Expression and integrated network analyses revealed functional divergence of NHX-type Na^+/H^+ exchanger genes in poplar

Fengxia Tian[1,2], Ermei Chang[2], Yu Li[2], Pei Sun[2], Jianjun Hu[2,*], Jin Zhang[2,*]

1. College of Life Science and Technology, Nanyang Normal University, Nanyang, Henan 473061, P.R. China;

2. State Key Laboratory of Tree Genetics and Breeding, Key Laboratory of Tree Breeding and Cultivation of the State Forestry Administration, Research Institute of Forestry, Chinese Academy of Forestry, Beijing 100091, P.R. China;

† These authors contributed equally to this work.

***** To whom correspondence should be addressed. Tel. +86 (10) 6288 8862. Fax. +86 (10) 6287 2015. Email: hujj@caf.ac.cn (J.H.) and zhang007jin@163.com (J.Z.).

Figure S1. Nucleotide and amino acid sequences of selected three *PtNHXs* (*-1*, *-6*, and *-8*) from three major groups cloned from *P. trichocarpa*.

The three cloned *PtNHXs* (*-1*, *-6*, and *-8*) showed 99.45%, 99.57%, and 99.54% identities with their corresponding CDS sequences from the *P. trichocarpa* genome database, respectively.

PtNHX1

Nucleotide

Identity = 99.45%

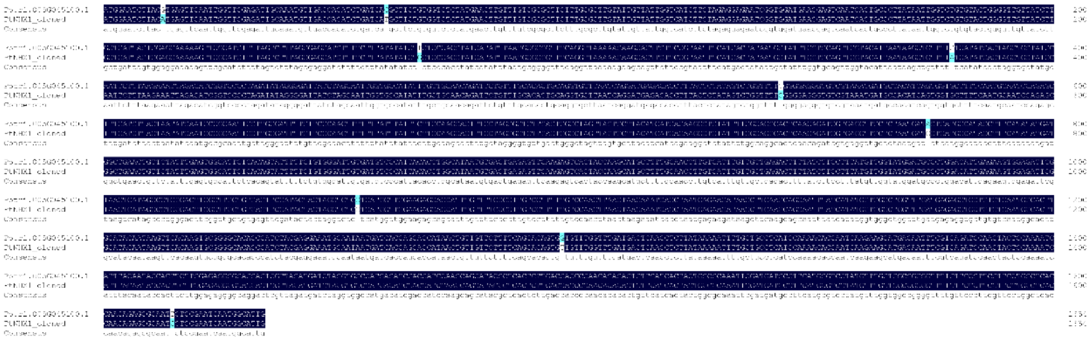

Amino acid

Identity = 98.9%

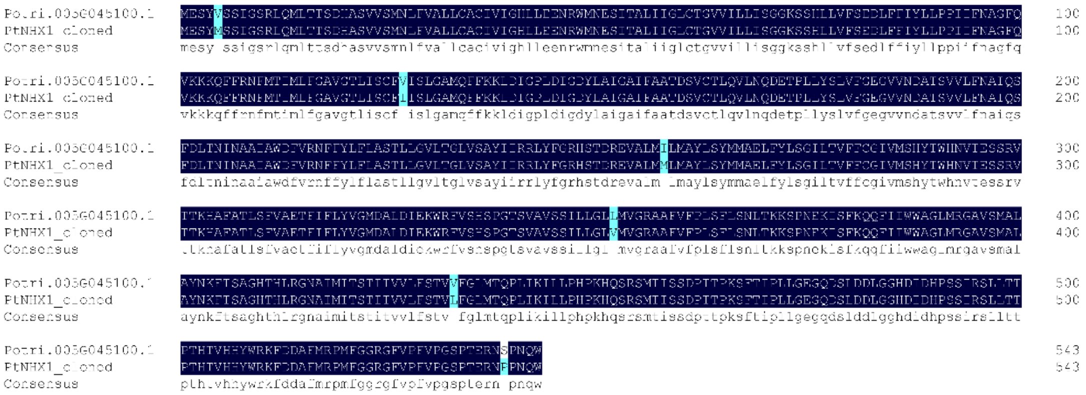

Figure S1 continue

PtNHX6

Nucleotide

Identity = 99.57%

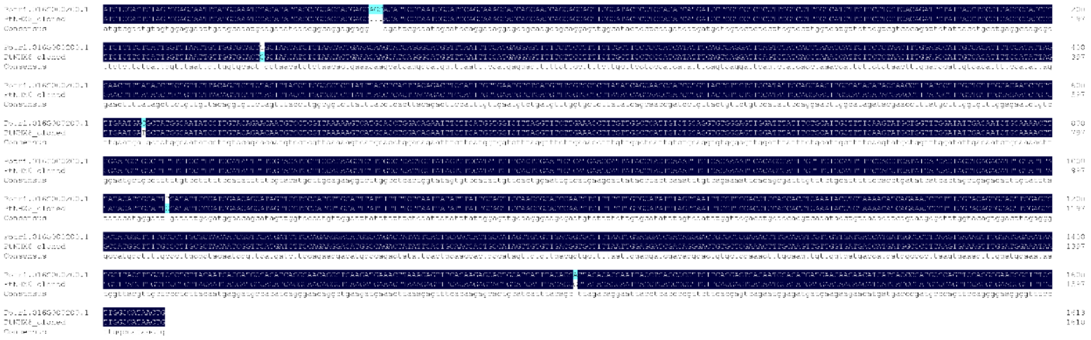

Amino acid

Identity = 99.81%

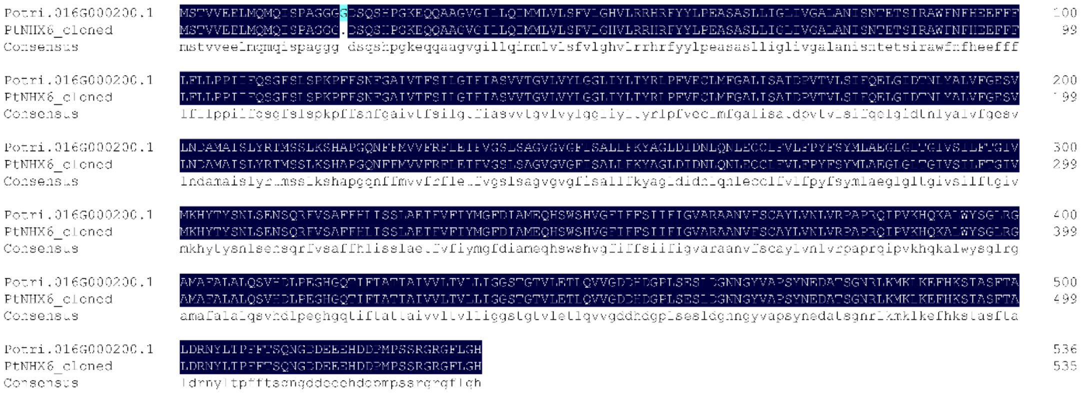

Figure S1 continue

PtNHX8

Nucleotide

Identity = 99.54%

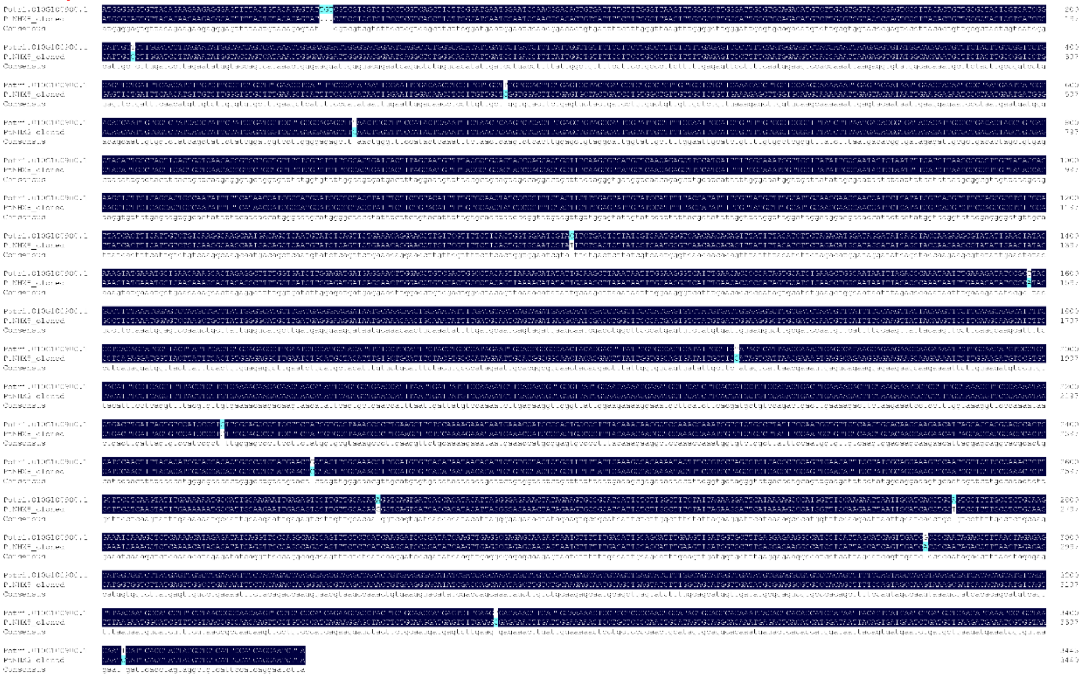

Amino acid

Identity = 99.39%

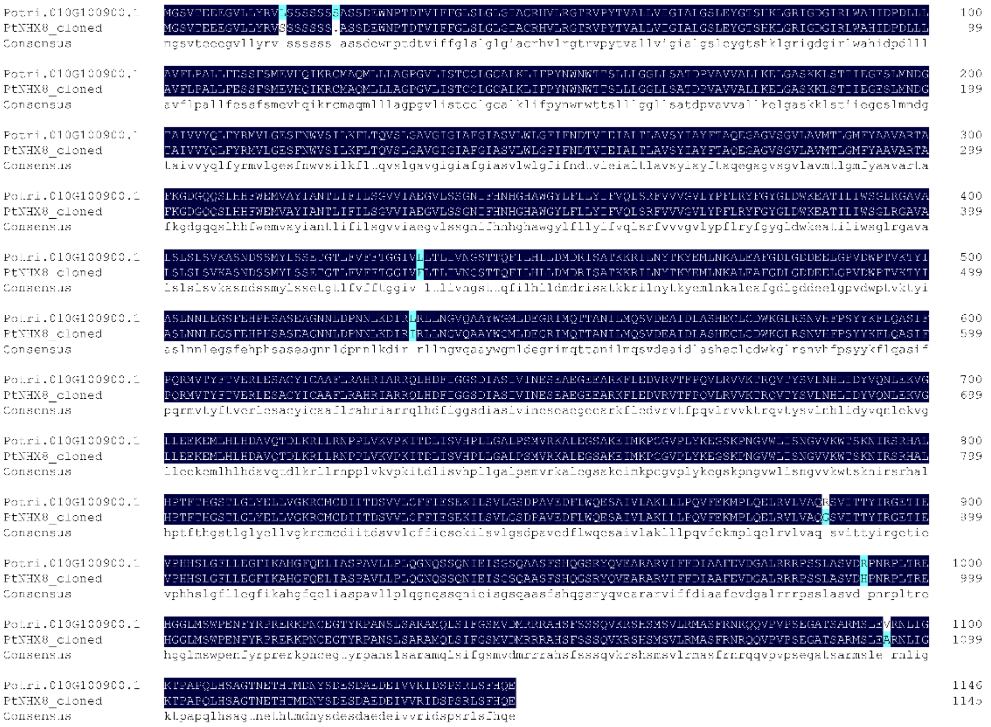

Figure S2. ***Arabidopsis NHX*** **map position and duplication analysis.**

(A) Schematic representation of *Arabidopsis* chromosomes showing the location of *AtNHX* genes.

(B) The duplicated *AtNHX* genes (red labelled) on *Arabidopsis* chromosomes.

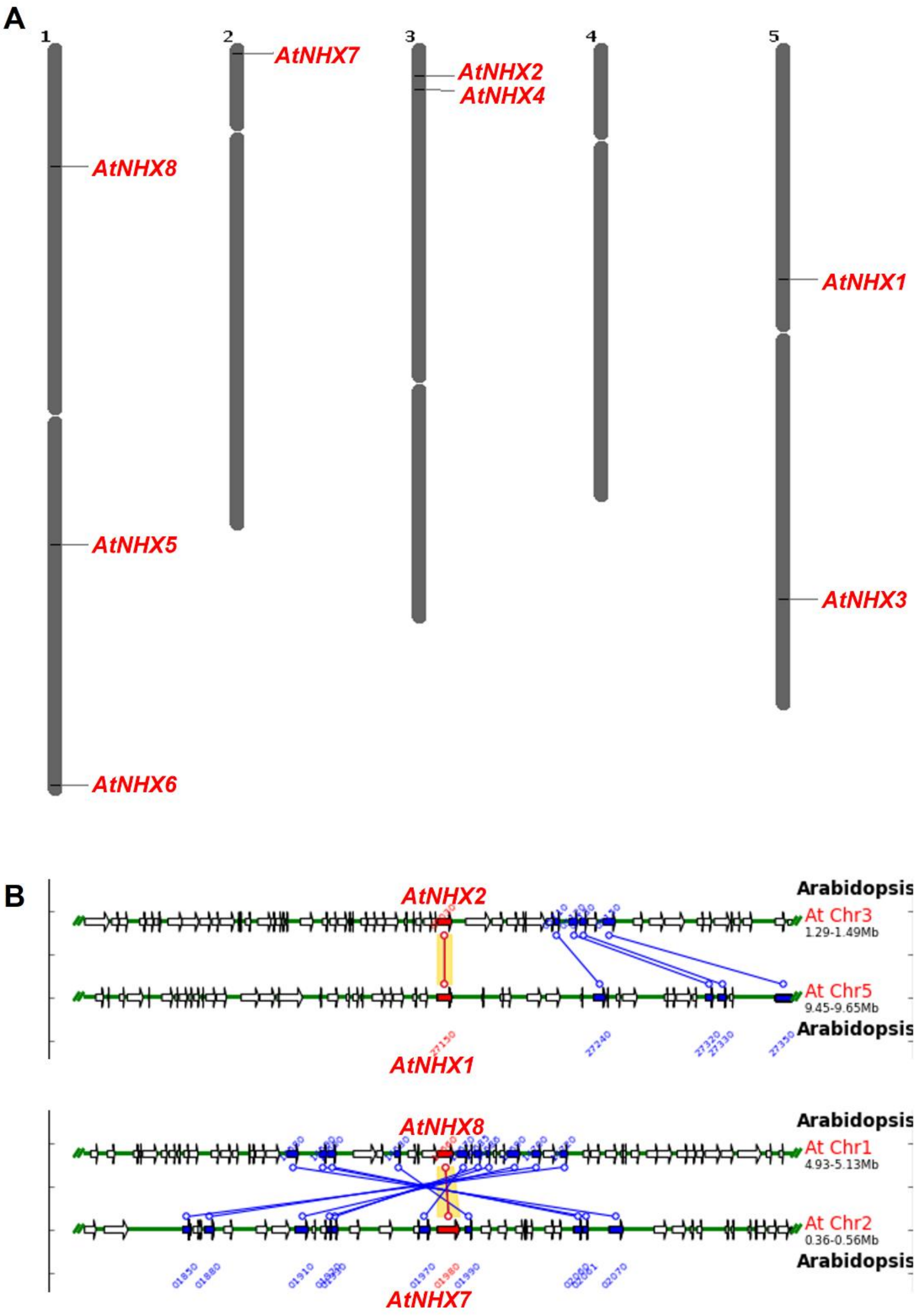

Figure S3. Sliding window analysis of duplicated *PtNHXs*.

The window size 60 and 90 bp were used for *K*a/*K*s analysis. The sequence identities and TM regions were shown in bottom of each figure.

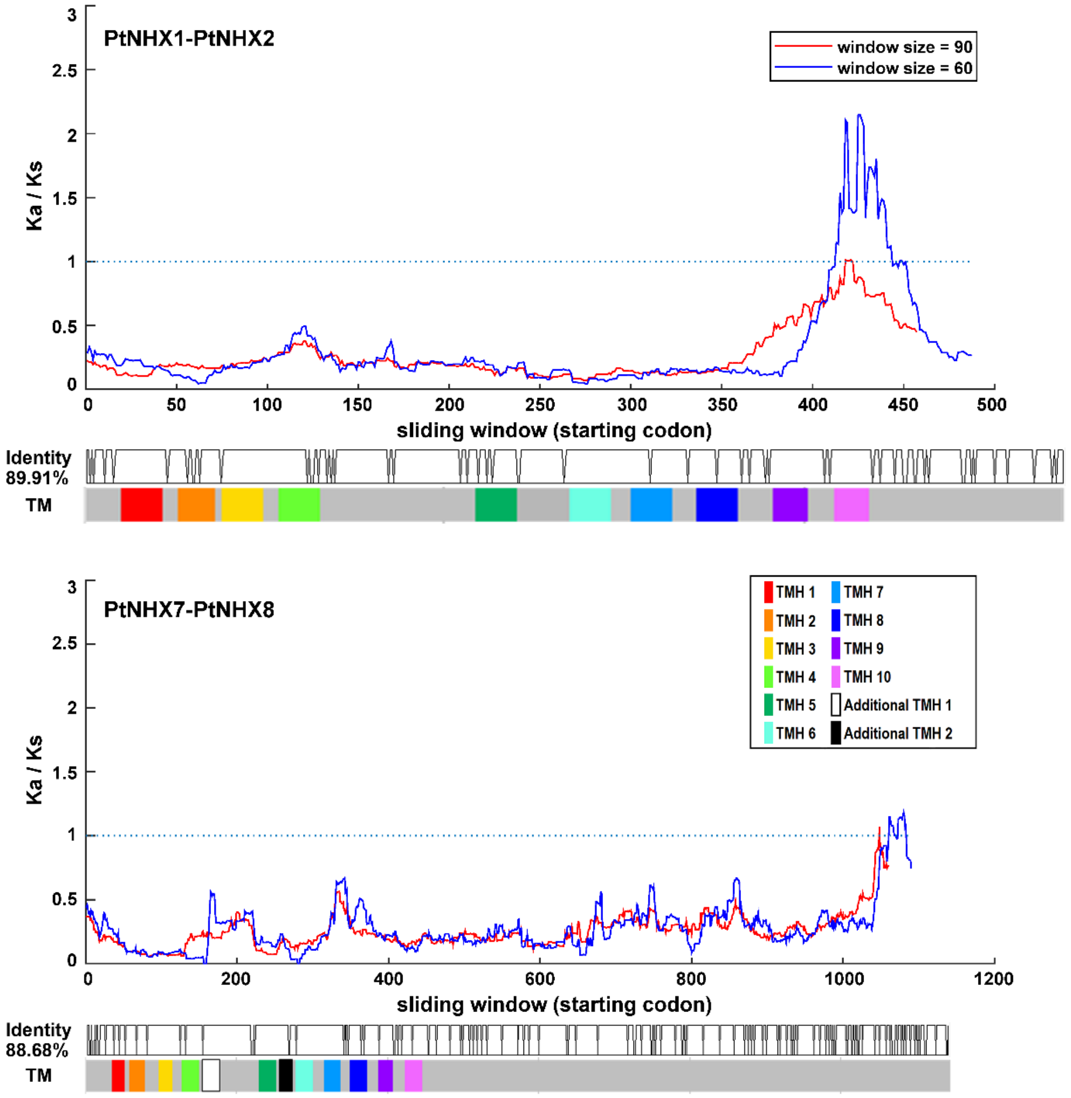

Figure S4. Details of motifs in AtNHXs and PtNHXs identified by MEME.

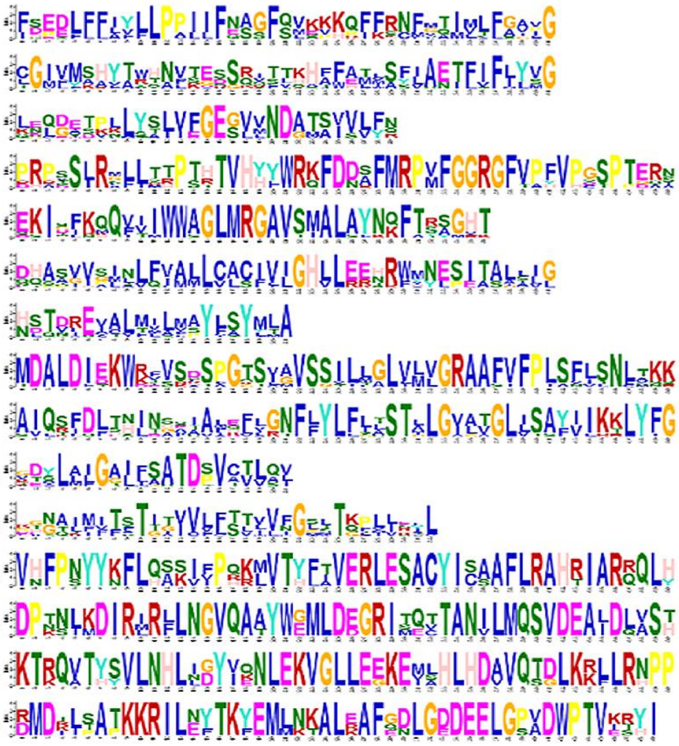

Motif	*E*-value	Sites	Width
1.	2.6e-307	16	41
2.	1.2e-293	16	41
3.	2.8e-202	16	29
4.	2.8e-173	7	50
5.	3.7e-179	9	36
6.	5.8e-200	12	41
7.	1.4e-136	16	21
8.	2.6e-190	9	50
9.	8.4e-147	9	50
10.	4.7e-125	16	21
11.	5.3e-137	16	32
12.	5.6e-075	4	50
13.	5.0e-073	4	50
14.	3.1e-071	4	50
15.	2.6e-067	4	49

Figure S5. Multiple sequence alignment and transmembrane region of PtNHXs.

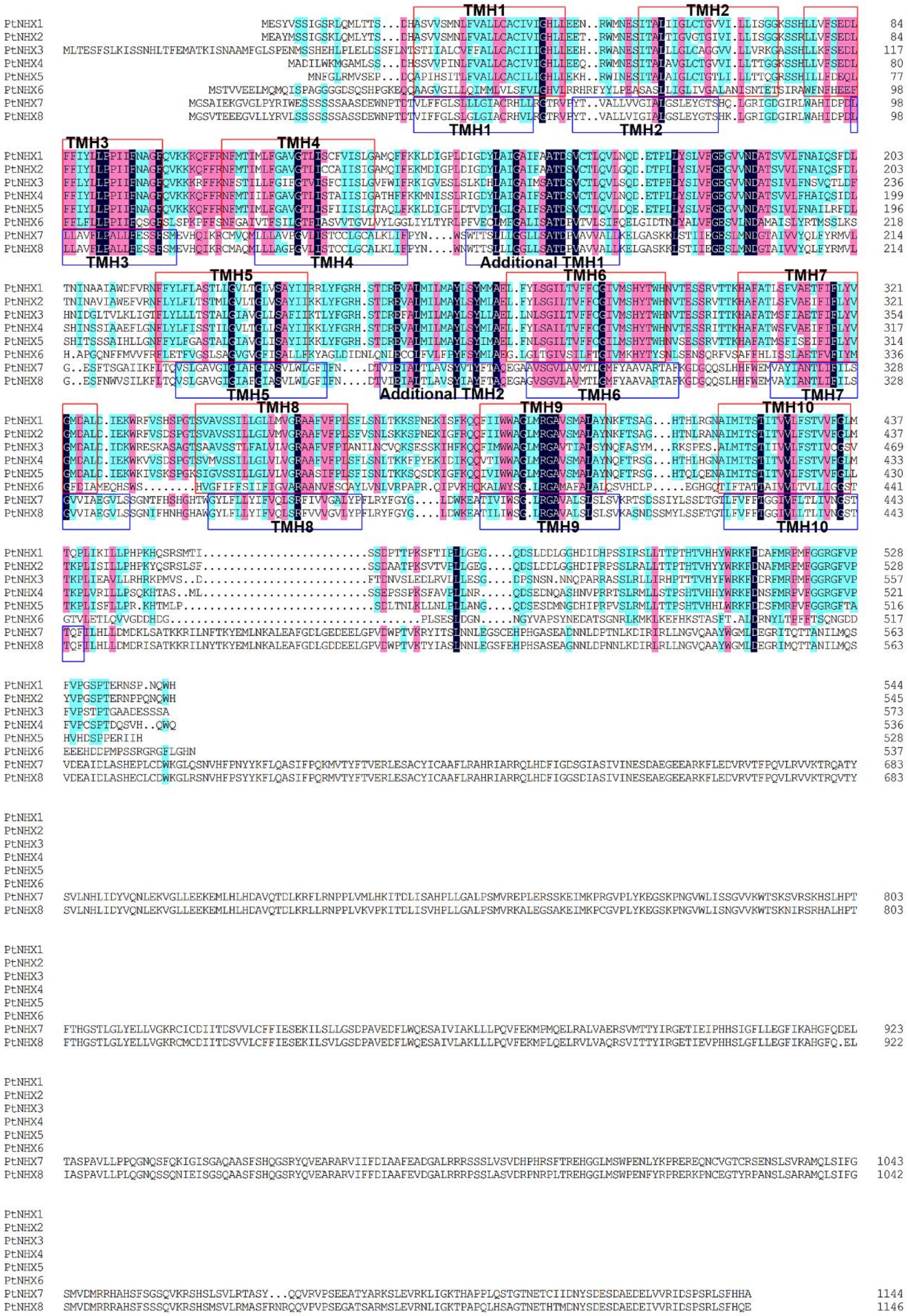

Figure S6. Details of secondary structure of PtNHX proteins.

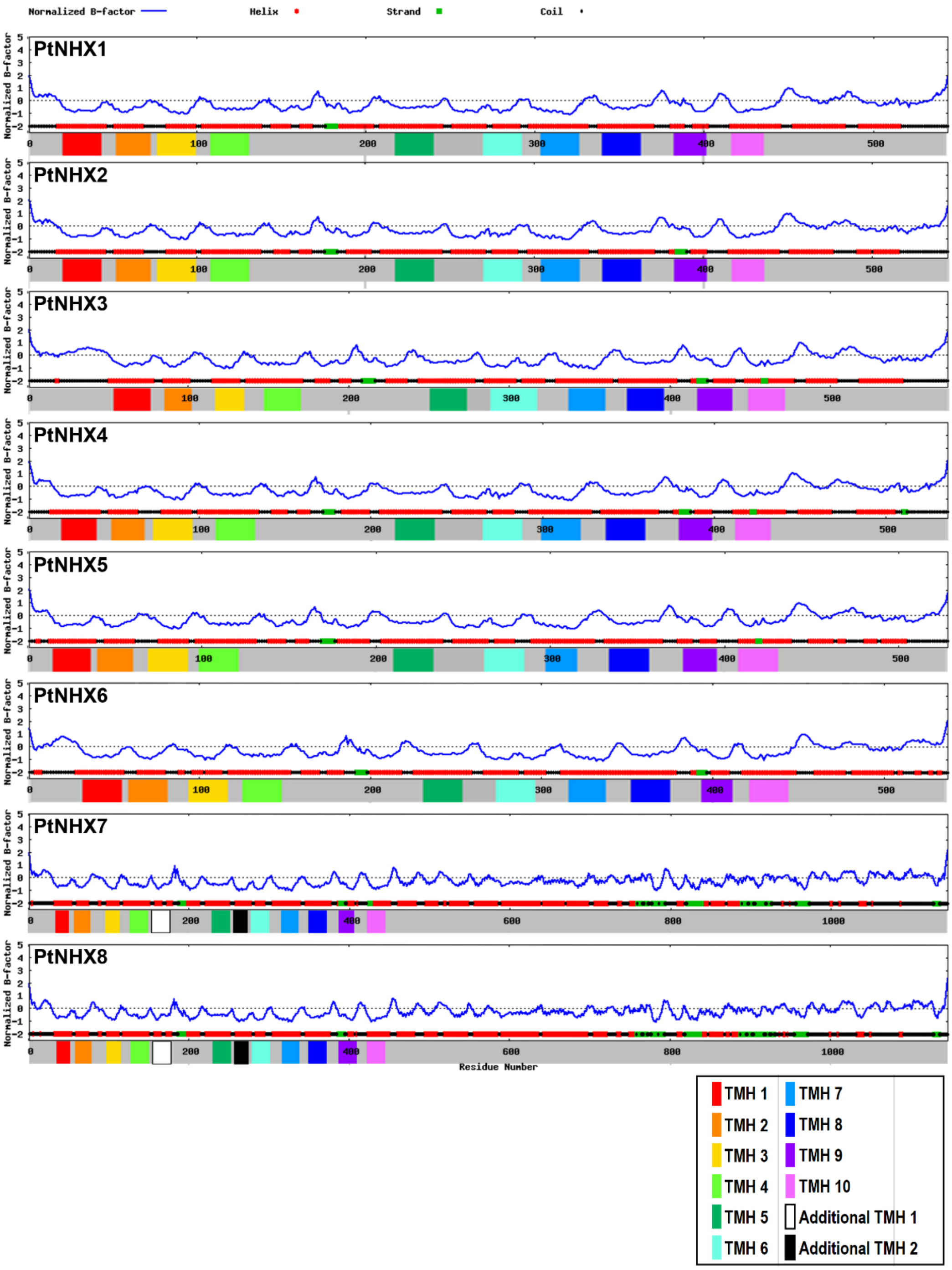

Figure S7. Expression patterns of *Arabidopsis NHX* genes across different tissues.

The data was obtained from AtGenExpress Visualization Tool (http://weigelworld.org/resources).

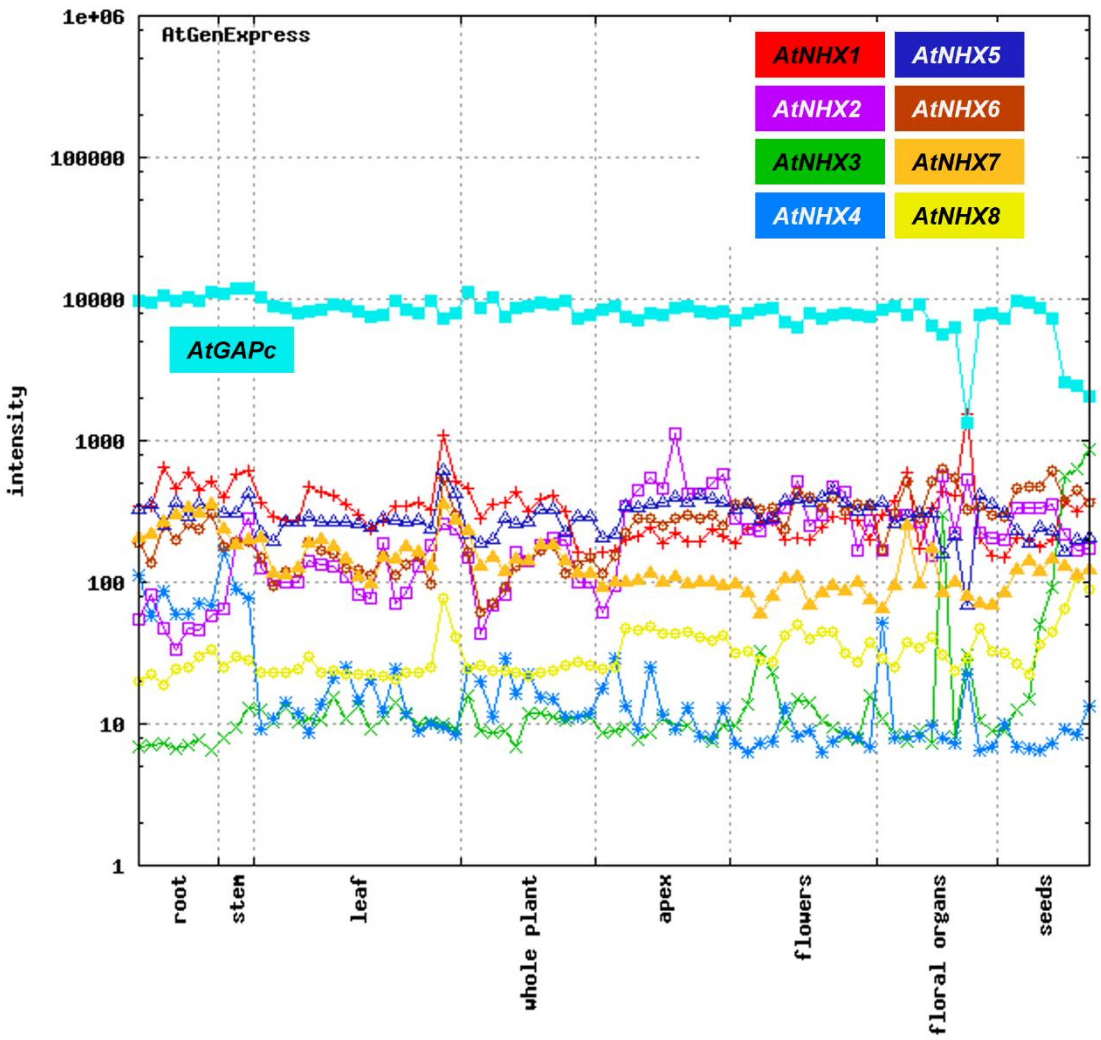

Figure S8. Expression patterns of *Arabidopsis NHX* genes under various abiotic stresses.

The microarray data was obtained from AtGenExpress Visualization Tool. Red means highly expression and green indicates low expression.

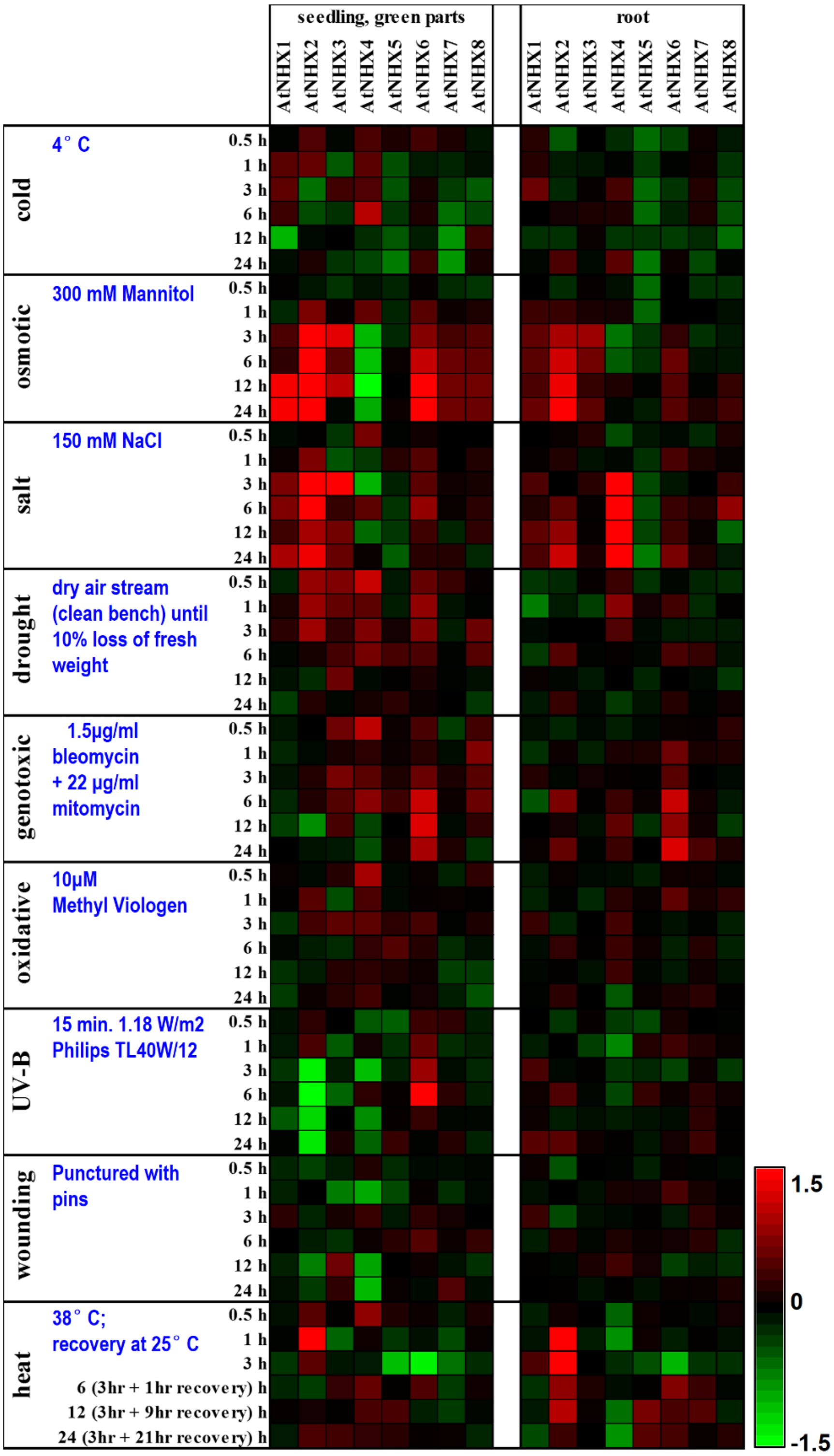

Figure S9. GO enrichment analysis of genes co-expressed with *PtNHXs*.

Enriched GO terms of all genes co-expressed with eight *PtNHXs*:

GO_acc	Type	Term	Gene	FDR	GO enrichment
GO:0080090	BP	regulation of primary metabolic process	73	1.4E-06	1.98
GO:0009889	BP	regulation of biosynthetic process	73	1.4E-06	1.99
GO:0016070	BP	RNA metabolic process	87	1.4E-06	1.84
GO:0006355	BP	regulation of transcription, DNA-dependent	73	1.4E-06	2.01
GO:0006350	BP	transcription	78	1.4E-06	1.96
GO:0006351	BP	transcription, DNA-dependent	78	1.4E-06	1.96
GO:0032774	BP	RNA biosynthetic process	78	1.4E-06	1.96
GO:0051252	BP	regulation of RNA metabolic process	73	1.4E-06	2.01
GO:0031326	BP	regulation of cellular biosynthetic process	73	1.4E-06	1.99
GO:0031323	BP	regulation of cellular metabolic process	74	1.4E-06	1.97
GO:0045449	BP	regulation of transcription	73	1.4E-06	2.01
GO:0010556	BP	regulation of macromolecule biosynthetic process	73	1.4E-06	1.99
GO:0019219	BP	regulation of nucleobase, nucleoside, nucleotide and nucleic acid metabolic process	73	1.4E-06	2.00
GO:0051171	BP	regulation of nitrogen compound metabolic process	73	1.4E-06	2.00
GO:0010468	BP	regulation of gene expression	73	1.6E-06	1.97
GO:0060255	BP	regulation of macromolecule metabolic process	73	1.8E-06	1.96
GO:0019222	BP	regulation of metabolic process	74	2.0E-06	1.94
GO:0050794	BP	regulation of cellular process	83	1.2E-03	1.56
GO:0006357	BP	regulation of transcription from RNA polymerase II promoter	6	1.2E-03	11.22
GO:0006139	BP	nucleobase, nucleoside, nucleotide and nucleic acid metabolic process	93	1.3E-03	1.51
GO:0006366	BP	transcription from RNA polymerase II promoter	7	1.5E-03	8.14
GO:0050789	BP	regulation of biological process	83	1.8E-03	1.54
GO:0065007	BP	biological regulation	83	6.0E-03	1.48
GO:0006807	BP	nitrogen compound metabolic process	98	1.0E-02	1.40
GO:0034645	BP	cellular macromolecule biosynthetic process	89	1.2E-02	1.42
GO:0009059	BP	macromolecule biosynthetic process	89	1.2E-02	1.41
GO:0010467	BP	gene expression	86	1.4E-02	1.42
GO:0044260	BP	cellular macromolecule metabolic process	166	1.5E-02	1.25
GO:0043170	BP	macromolecule metabolic process	178	4.3E-02	1.21
GO:0005515	MF	protein binding	160	1.3E-09	1.70
GO:0005488	MF	binding	365	2.0E-09	1.25
GO:0003676	MF	nucleic acid binding	125	8.9E-07	1.66
GO:0030528	MF	transcription regulator activity	57	4.4E-06	2.21
GO:0008270	MF	zinc ion binding	44	2.5E-05	2.36
GO:0003702	MF	RNA polymerase II transcription factor activity	8	1.0E-03	8.19
GO:0016455	MF	RNA polymerase II transcription mediator activity	6	1.8E-03	11.73
GO:0043565	MF	sequence-specific DNA binding	29	2.5E-03	2.30
GO:0016251	MF	general RNA polymerase II transcription factor activity	6	3.1E-03	9.93
GO:0003700	MF	transcription factor activity	35	4.7E-03	2.01
GO:0003677	MF	DNA binding	77	6.0E-03	1.52
GO:0016592	CC	mediator complex	6	3.8E-03	11.73
GO:0044428	CC	nuclear part	10	7.6E-03	4.06
GO:0005654	CC	nucleoplasm	7	7.6E-03	5.90
GO:0005634	CC	nucleus	41	7.6E-03	1.85
GO:0044451	CC	nucleoplasm part	7	7.6E-03	5.90
GO:0031981	CC	nuclear lumen	7	1.3E-02	5.19
GO:0043231	CC	intracellular membrane-bounded organelle	49	2.8E-02	1.55
GO:0043233	CC	organelle lumen	7	2.8E-02	4.07
GO:0043227	CC	membrane-bounded organelle	49	2.8E-02	1.54
GO:0070013	CC	intracellular organelle lumen	7	2.8E-02	4.07
GO:0031974	CC	membrane-enclosed lumen	7	4.6E-02	3.63

Figure S9 continue

Enriched BP terms of all genes co-expressed with eight *PtNHXs*:

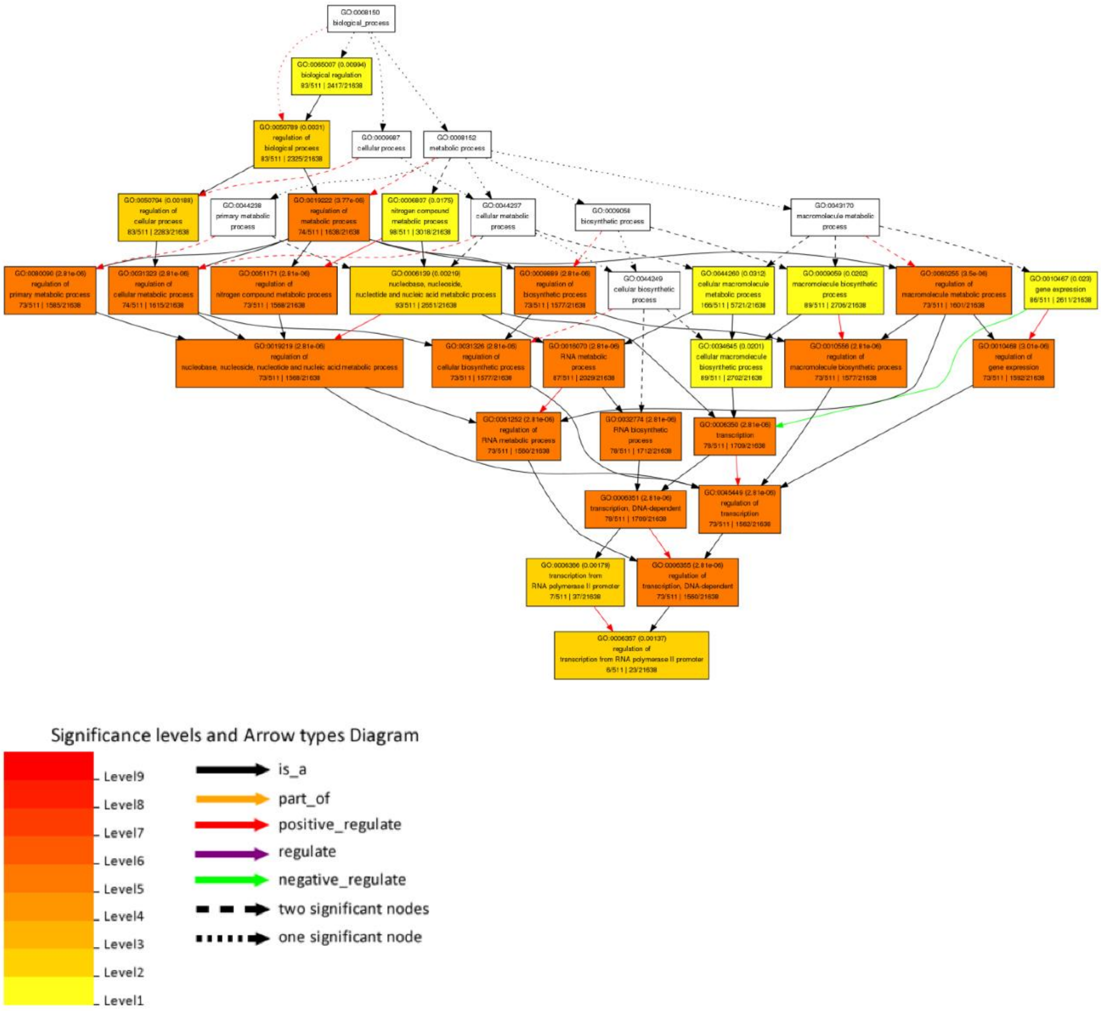

Figure S9 continue

Enriched MF terms of all genes co-expressed with eight *PtNHXs*:

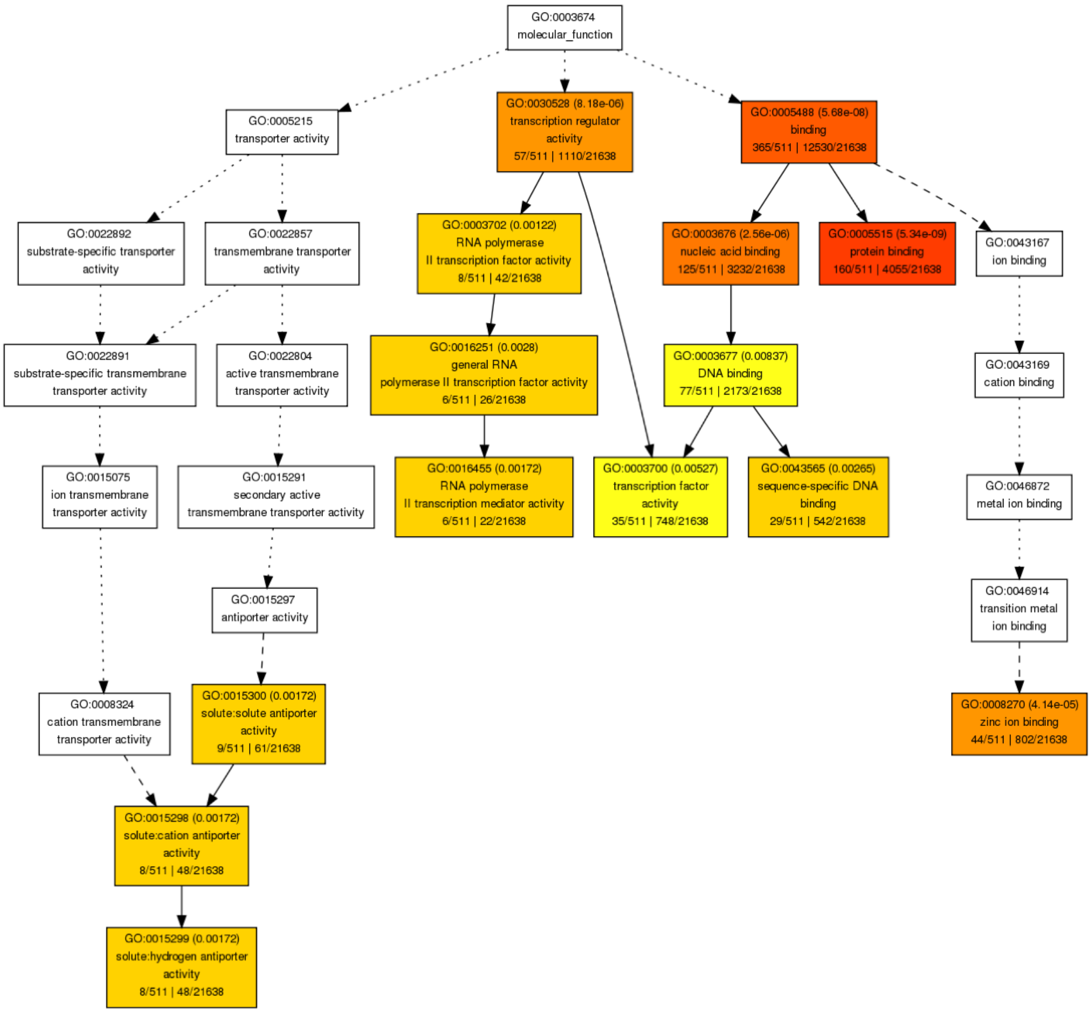

Figure S9 continue

Enriched CC terms of all genes co-expressed with eight *PtNHXs*:

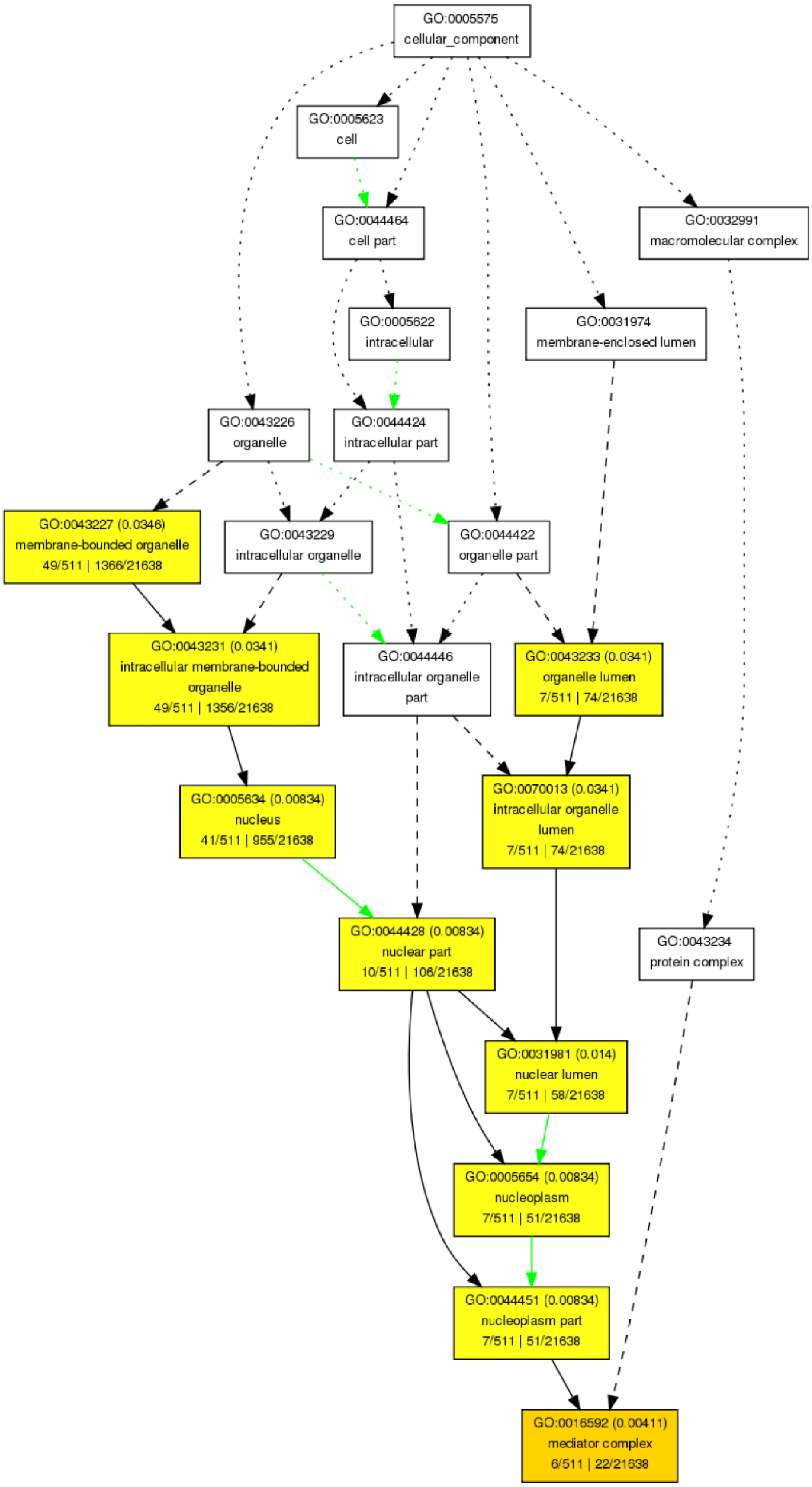

Figure S9 continue

Enriched GO terms of genes co-expressed with *PtNHX3*:

GO_acc	Type	Term	Gene	FDR	GO enrichment
GO:0080090	BP	regulation of primary metabolic process	13	7.2E-04	3.62
GO:0045449	BP	regulation of transcription	13	7.2E-04	3.68
GO:0019219	BP	regulation of nucleobase, nucleoside, nucleotide and nucleic acid metabolic process	13	7.2E-04	3.66
GO:0031326	BP	regulation of cellular biosynthetic process	13	7.2E-04	3.64
GO:0010468	BP	regulation of gene expression	13	7.2E-04	3.61
GO:0060255	BP	regulation of macromolecule metabolic process	13	7.2E-04	3.59
GO:0009889	BP	regulation of biosynthetic process	13	7.2E-04	3.64
GO:0051171	BP	regulation of nitrogen compound metabolic process	13	7.2E-04	3.66
GO:0051252	BP	regulation of RNA metabolic process	13	7.2E-04	3.68
GO:0006355	BP	regulation of transcription, DNA-dependent	13	7.2E-04	3.68
GO:0010556	BP	regulation of macromolecule biosynthetic process	13	7.2E-04	3.64
GO:0031323	BP	regulation of cellular metabolic process	13	7.3E-04	3.55
GO:0019222	BP	regulation of metabolic process	13	7.7E-04	3.50
GO:0032774	BP	RNA biosynthetic process	13	9.9E-04	3.35
GO:0006350	BP	transcription	13	9.9E-04	3.36
GO:0006351	BP	transcription, DNA-dependent	13	9.9E-04	3.36
GO:0016070	BP	RNA metabolic process	14	1.3E-03	3.05
GO:0050794	BP	regulation of cellular process	13	1.4E-02	2.51
GO:0010467	BP	gene expression	14	1.5E-02	2.37
GO:0050789	BP	regulation of biological process	13	1.5E-02	2.47
GO:0006139	BP	nucleobase, nucleoside, nucleotide and nucleic acid metabolic process	14	1.6E-02	2.33
GO:0006807	BP	nitrogen compound metabolic process	15	1.8E-02	2.19
GO:0065007	BP	biological regulation	13	1.9E-02	2.38
GO:0034645	BP	cellular macromolecule biosynthetic process	13	4.4E-02	2.12
GO:0009059	BP	macromolecule biosynthetic process	13	4.4E-02	2.12
GO:0008152	BP	metabolic process	35	4.4E-02	1.36
GO:0043170	BP	macromolecule metabolic process	23	4.9E-02	1.60
GO:0030528	MF	transcription regulator activity	11	1.3E-03	4.38
GO:0003700	MF	transcription factor activity	9	1.3E-03	5.31
GO:0043565	MF	sequence-specific DNA binding	8	1.3E-03	6.52
GO:0003677	MF	DNA binding	15	1.4E-03	3.05

Figure S9 continue

Enriched BP terms of genes co-expressed with *PtNHX3*:

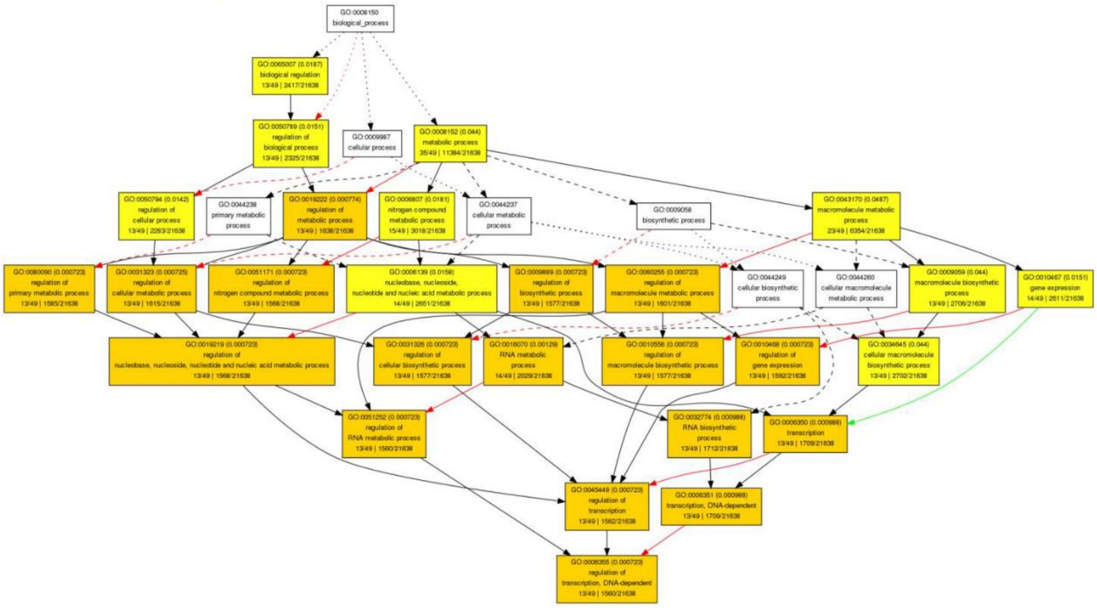

Figure S9 continue

Enriched MF terms of genes co-expressed with *PtNHX3*:

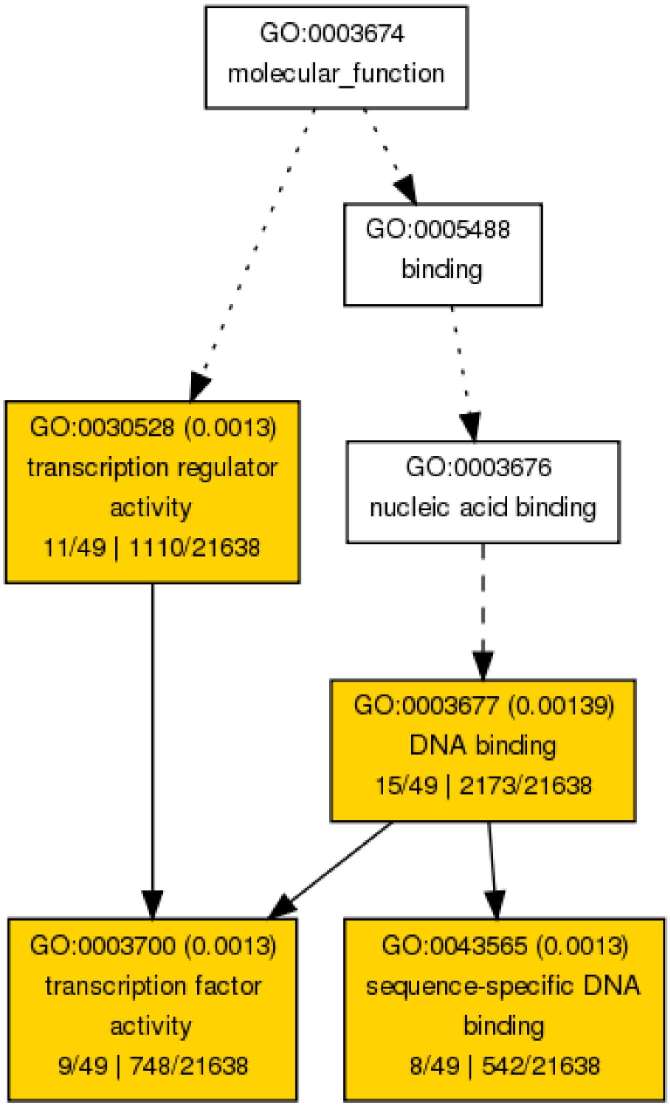

Figure S9 continue

Enriched GO terms of genes co-expressed with *PtNHX6*:

GO_acc	Type	Term	Gene	FDR	GO enrichment
GO:0005515	MF	protein binding	51	2.1E-06	2.16
GO:0005488	MF	binding	98	1.7E-04	1.34
GO:0008270	MF	zinc ion binding	16	9.6E-04	3.43
GO:0003676	MF	nucleic acid binding	33	2.7E-02	1.75

Enriched MF terms of genes co-expressed with *PtNHX6*:

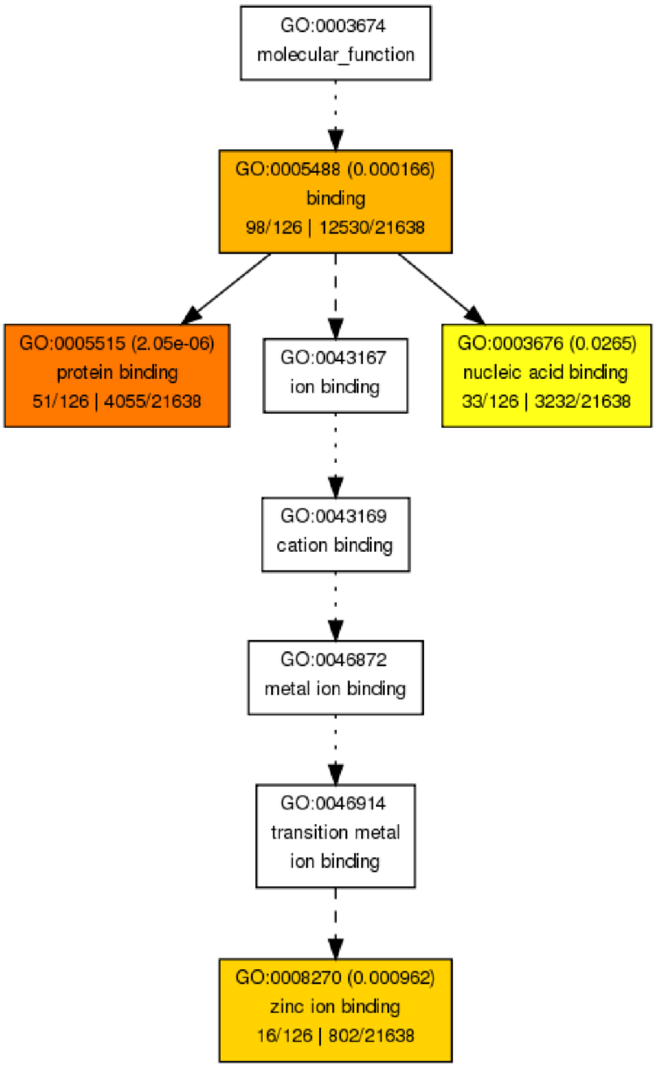

Figure S9 continue

Enriched GO terms of genes co-expressed with *PtNHX8*:

GO_acc	Type	Term	Gene	FDR	GO enrichment
GO:0032774	BP	RNA biosynthetic process	44	3.0E-04	2.06
GO:0031326	BP	regulation of cellular biosynthetic process	41	3.0E-04	2.08
GO:0045449	BP	regulation of transcription	41	3.0E-04	2.10
GO:0019219	BP	regulation of nucleobase, nucleoside, nucleotide and nucleic acid metabolic process	41	3.0E-04	2.10
GO:0009889	BP	regulation of biosynthetic process	41	3.0E-04	2.08
GO:0051171	BP	regulation of nitrogen compound metabolic process	41	3.0E-04	2.10
GO:0051252	BP	regulation of RNA metabolic process	41	3.0E-04	2.11
GO:0006355	BP	regulation of transcription, DNA-dependent	41	3.0E-04	2.11
GO:0010556	BP	regulation of macromolecule biosynthetic process	41	3.0E-04	2.08
GO:0006350	BP	transcription	44	3.0E-04	2.06
GO:0006351	BP	transcription, DNA-dependent	44	3.0E-04	2.06
GO:0080090	BP	regulation of primary metabolic process	41	3.1E-04	2.07
GO:0010468	BP	regulation of gene expression	41	3.2E-04	2.06
GO:0060255	BP	regulation of macromolecule metabolic process	41	3.4E-04	2.05
GO:0031323	BP	regulation of cellular metabolic process	41	3.7E-04	2.03
GO:0016070	BP	RNA metabolic process	48	3.7E-04	1.90
GO:0019222	BP	regulation of metabolic process	41	4.7E-04	2.01
GO:0006366	BP	transcription from RNA polymerase II promoter	5	3.5E-03	10.83
GO:0006139	BP	nucleobase, nucleoside, nucleotide and nucleic acid metabolic process	53	8.3E-03	1.60
GO:0034645	BP	cellular macromolecule biosynthetic process	52	2.1E-02	1.54
GO:0009059	BP	macromolecule biosynthetic process	52	2.1E-02	1.54
GO:0050794	BP	regulation of cellular process	45	2.8E-02	1.58
GO:0050789	BP	regulation of biological process	45	3.8E-02	1.55
GO:0006807	BP	nitrogen compound metabolic process	55	4.2E-02	1.46
GO:0003676	MF	nucleic acid binding	74	3.2E-05	1.83
GO:0005488	MF	binding	194	1.5E-04	1.24
GO:0005515	MF	protein binding	83	1.5E-04	1.64
GO:0030528	MF	transcription regulator activity	33	3.4E-04	2.38
GO:0008270	MF	zinc ion binding	24	5.2E-03	2.40
GO:0003677	MF	DNA binding	47	7.2E-03	1.73
GO:0003702	MF	RNA polymerase II transcription factor activity	5	1.1E-02	9.54
GO:0005654	CC	nucleoplasm	5	2.0E-02	7.86
GO:0005634	CC	nucleus	25	2.0E-02	2.10
GO:0044451	CC	nucleoplasm part	5	2.0E-02	7.86
GO:0031981	CC	nuclear lumen	5	2.6E-02	6.91
GO:0043233	CC	organelle lumen	5	4.0E-02	5.41
GO:0044428	CC	nuclear part	6	4.0E-02	4.54
GO:0070013	CC	intracellular organelle lumen	5	4.0E-02	5.41

Figure S9 continue

Enriched BP terms of genes co-expressed with *PtNHX8*:

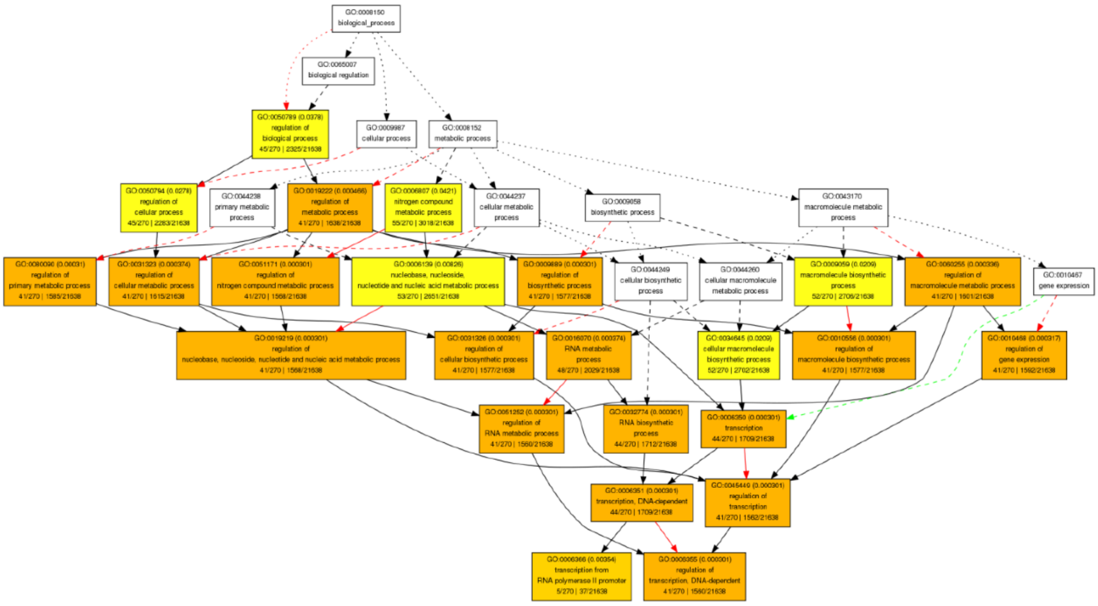

Figure S9 continue

Enriched MF terms of genes co-expressed with *PtNHX8*:

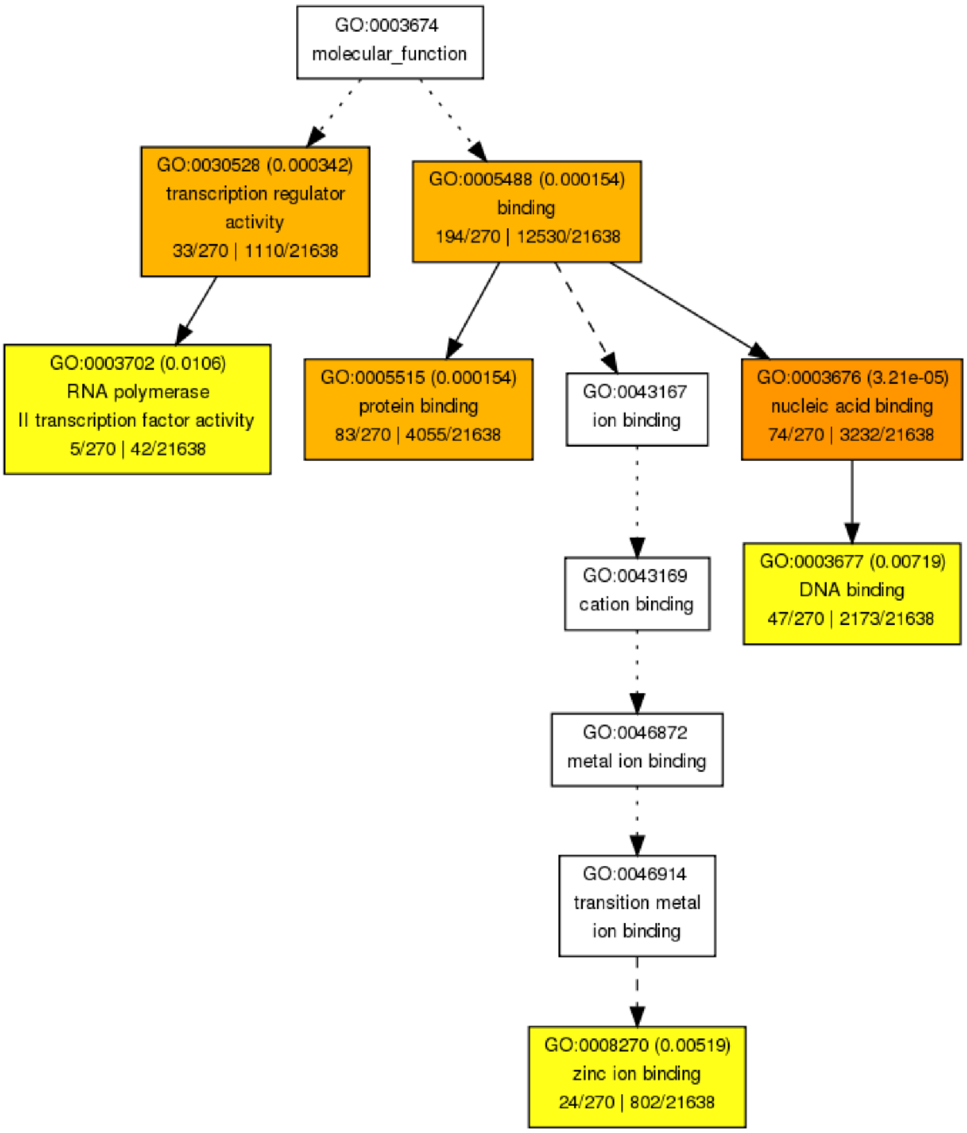

Figure S9 continue

Enriched CC terms of genes co-expressed with *PtNHX8*:

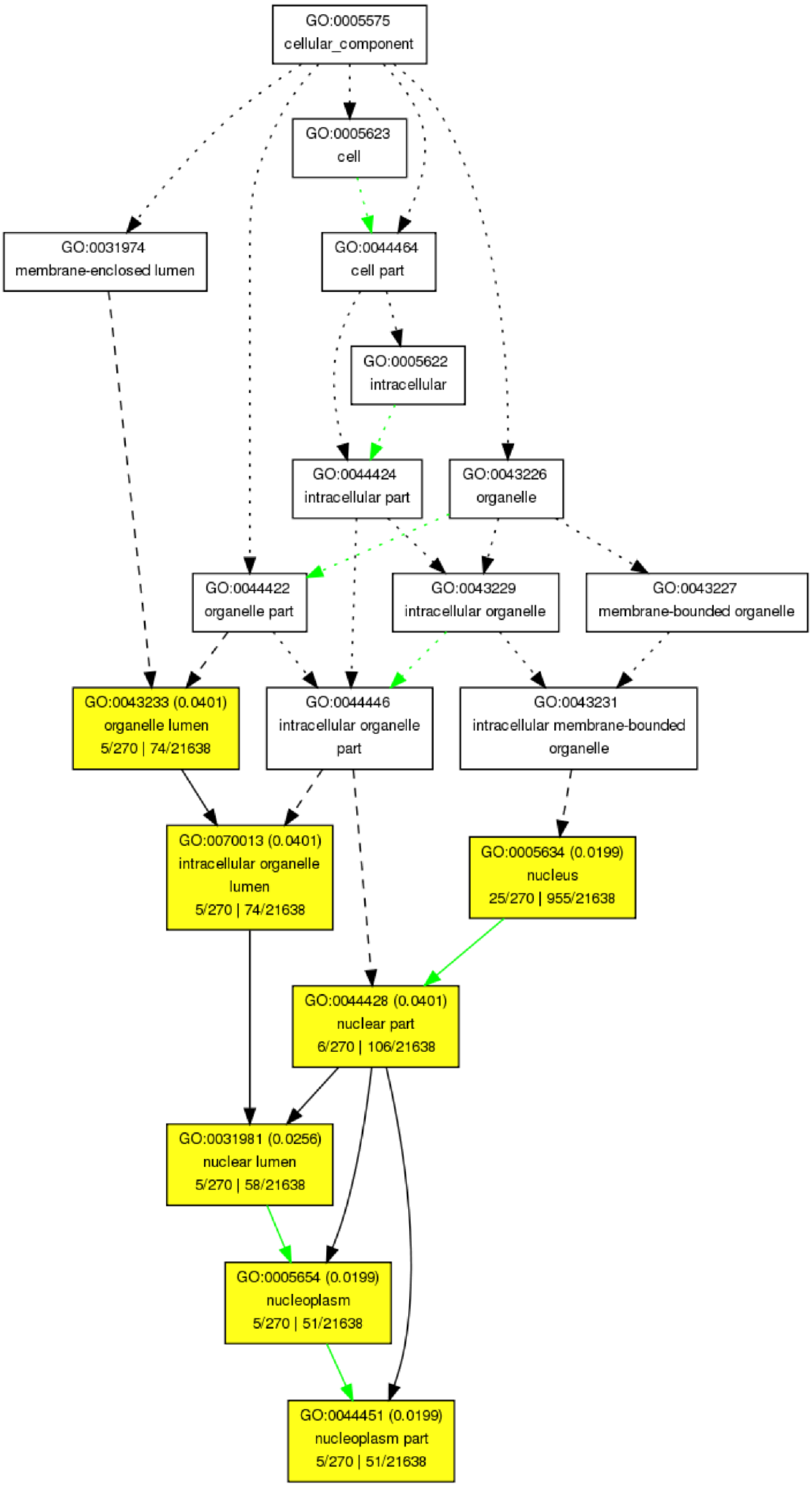

Figure S10. Co-expression network of *Arabidopsis NHX* genes.

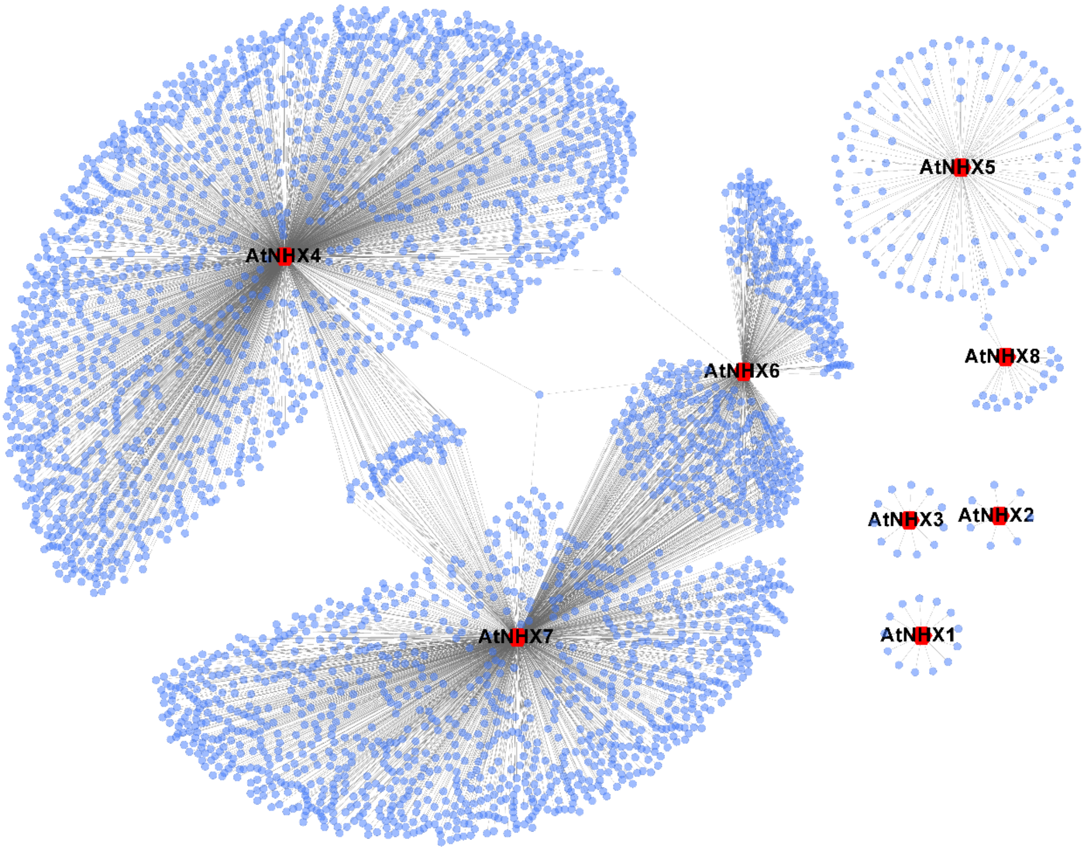

Figure S11. GO enrichment analysis of PtNHX PPI network.

Enriched GO terms of genes in the PtNHX PPI network:

GO_acc	Type	Term	Gene	FDR	GO enrichment
GO:0055085	BP	transmembrane transport	28	1.6E-18	10.05
GO:0006810	BP	transport	35	1.6E-17	5.99
GO:0051234	BP	establishment of localization	35	1.6E-17	5.99
GO:0051179	BP	localization	35	1.8E-17	5.92
GO:0006812	BP	cation transport	21	2.7E-17	15.10
GO:0006811	BP	ion transport	22	3.1E-17	13.41
GO:0043038	BP	amino acid activation	12	3.0E-15	48.08
GO:0043039	BP	tRNA aminoacylation	12	3.0E-15	48.08
GO:0006418	BP	tRNA aminoacylation for protein translation	12	3.0E-15	48.73
GO:0006399	BP	tRNA metabolic process	12	5.2E-12	24.20
GO:0034660	BP	ncRNA metabolic process	12	5.5E-11	19.39
GO:0006520	BP	cellular amino acid metabolic process	12	1.4E-08	11.63
GO:0044106	BP	cellular amine metabolic process	12	2.2E-08	11.10
GO:0006519	BP	cellular amino acid and derivative metabolic process	12	4.0E-08	10.45
GO:0009308	BP	amine metabolic process	12	7.2E-08	9.83
GO:0034641	BP	cellular nitrogen compound metabolic process	12	8.1E-07	7.81
GO:0009987	BP	cellular process	55	1.1E-06	1.69
GO:0019752	BP	carboxylic acid metabolic process	12	1.5E-06	7.23
GO:0042180	BP	cellular ketone metabolic process	12	1.5E-06	7.20
GO:0006082	BP	organic acid metabolic process	12	1.5E-06	7.21
GO:0043436	BP	oxoacid metabolic process	12	1.5E-06	7.23
GO:0006412	BP	translation	12	2.8E-05	5.38
GO:0016192	BP	vesicle-mediated transport	7	2.9E-05	11.95
GO:0006886	BP	intracellular protein transport	7	4.9E-05	10.96
GO:0070727	BP	cellular macromolecule localization	7	6.2E-05	10.41
GO:0034613	BP	cellular protein localization	7	6.2E-05	10.41
GO:0046907	BP	intracellular transport	7	1.4E-04	9.11
GO:0045184	BP	establishment of protein localization	7	3.9E-04	7.65
GO:0015031	BP	protein transport	7	3.9E-04	7.65
GO:0051649	BP	establishment of localization in cell	7	4.3E-04	7.49
GO:0008104	BP	protein localization	7	5.8E-04	7.08
GO:0051641	BP	cellular localization	7	5.8E-04	7.04
GO:0033036	BP	macromolecule localization	7	6.9E-04	6.81
GO:0044281	BP	small molecule metabolic process	12	1.8E-03	3.34
GO:0007165	BP	signal transduction	8	2.5E-03	4.69
GO:0007264	BP	small GTPase mediated signal transduction	5	3.5E-03	7.95
GO:0007242	BP	intracellular signaling cascade	5	3.9E-03	7.71
GO:0023046	BP	signaling process	8	4.8E-03	4.17
GO:0023060	BP	signal transmission	8	4.8E-03	4.17
GO:0023052	BP	signaling	8	4.4E-02	2.89
GO:0015299	MF	solute:hydrogen antiporter activity	20	6.0E-33	125.22
GO:0015298	MF	solute:cation antiporter activity	20	6.0E-33	125.22
GO:0015300	MF	solute:solute antiporter activity	20	2.2E-31	98.53
GO:0015297	MF	antiporter activity	20	2.9E-25	43.87
GO:0015291	MF	secondary active transmembrane transporter activity	20	2.6E-24	38.28
GO:0008324	MF	cation transmembrane transporter activity	24	2.6E-24	22.33
GO:0015075	MF	ion transmembrane transporter activity	25	1.8E-21	15.06
GO:0022891	MF	substrate-specific transmembrane transporter activity	25	2.7E-20	13.34
GO:0022892	MF	substrate-specific transporter activity	25	4.4E-19	11.76
GO:0022804	MF	active transmembrane transporter activity	20	1.0E-17	16.33
GO:0022857	MF	transmembrane transporter activity	25	3.9E-16	8.68
GO:0004812	MF	aminoacyl-tRNA ligase activity	12	9.3E-16	48.08
GO:0016875	MF	ligase activity, forming carbon-oxygen bonds	12	9.3E-16	47.45
GO:0016876	MF	ligase activity, forming aminoacyl-tRNA and related compounds	12	9.3E-16	47.45
GO:0005215	MF	transporter activity	25	1.6E-13	6.54
GO:0016874	MF	ligase activity	12	2.8E-08	9.85
GO:0044425	CC	membrane part	34	8.8E-18	6.33
GO:0044464	CC	cell part	56	1.3E-17	2.87
GO:0005623	CC	cell	56	1.3E-17	2.87
GO:0016021	CC	integral to membrane	26	1.7E-14	6.97
GO:0016020	CC	membrane	39	1.7E-14	3.89
GO:0031224	CC	intrinsic to membrane	27	8.7E-14	6.07
GO:0005737	CC	cytoplasm	22	4.6E-09	4.75
GO:0030117	CC	membrane coat	7	1.1E-08	37.57
GO:0048475	CC	coated membrane	7	1.1E-08	37.57
GO:0044424	CC	intracellular part	22	3.0E-04	2.44
GO:0005622	CC	intracellular	25	3.1E-04	2.23

Figure S11 continue

Enriched BP terms of genes in the PtNHX PPI network:

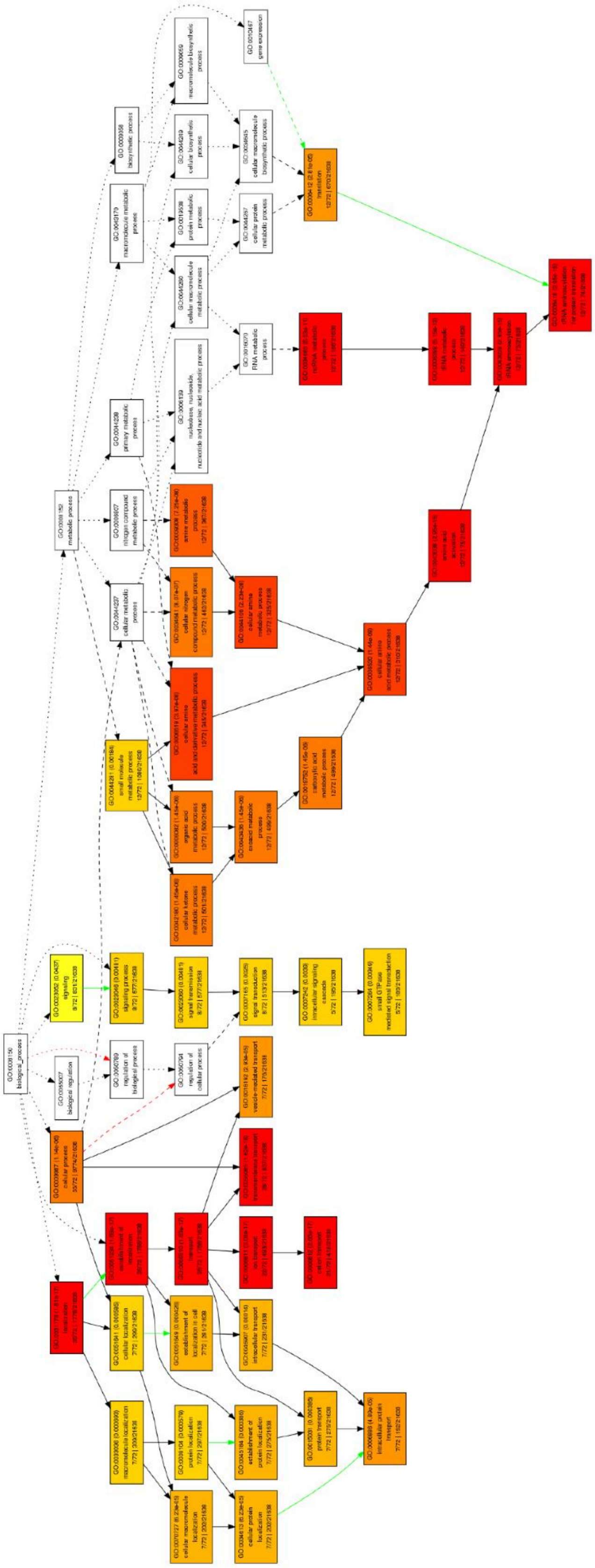

Figure S11 continue

Enriched MF terms of genes in the PtNHX PPI network:

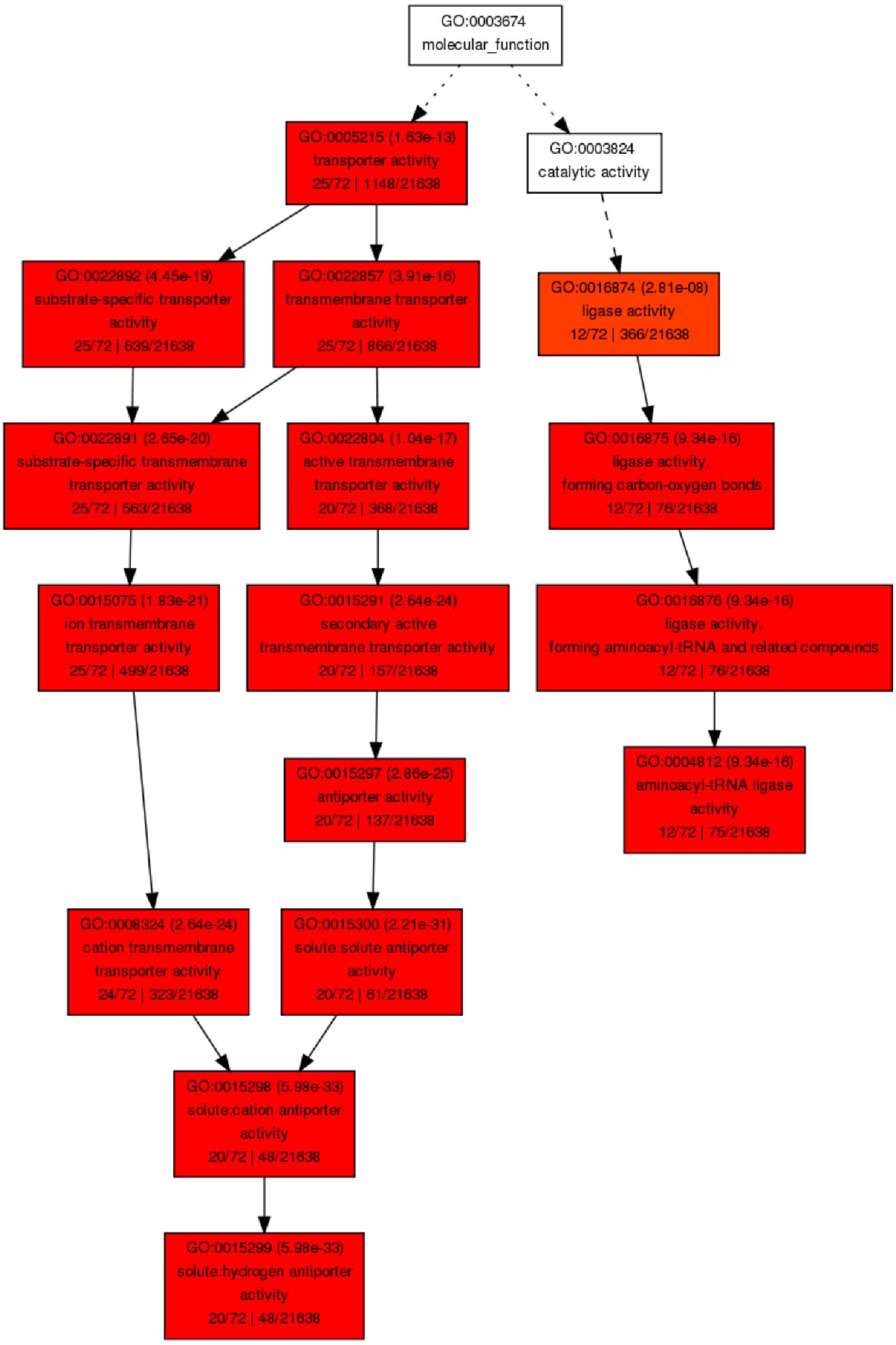

Figure S11 continue

Enriched CC terms of genes in the PtNHX PPI network:

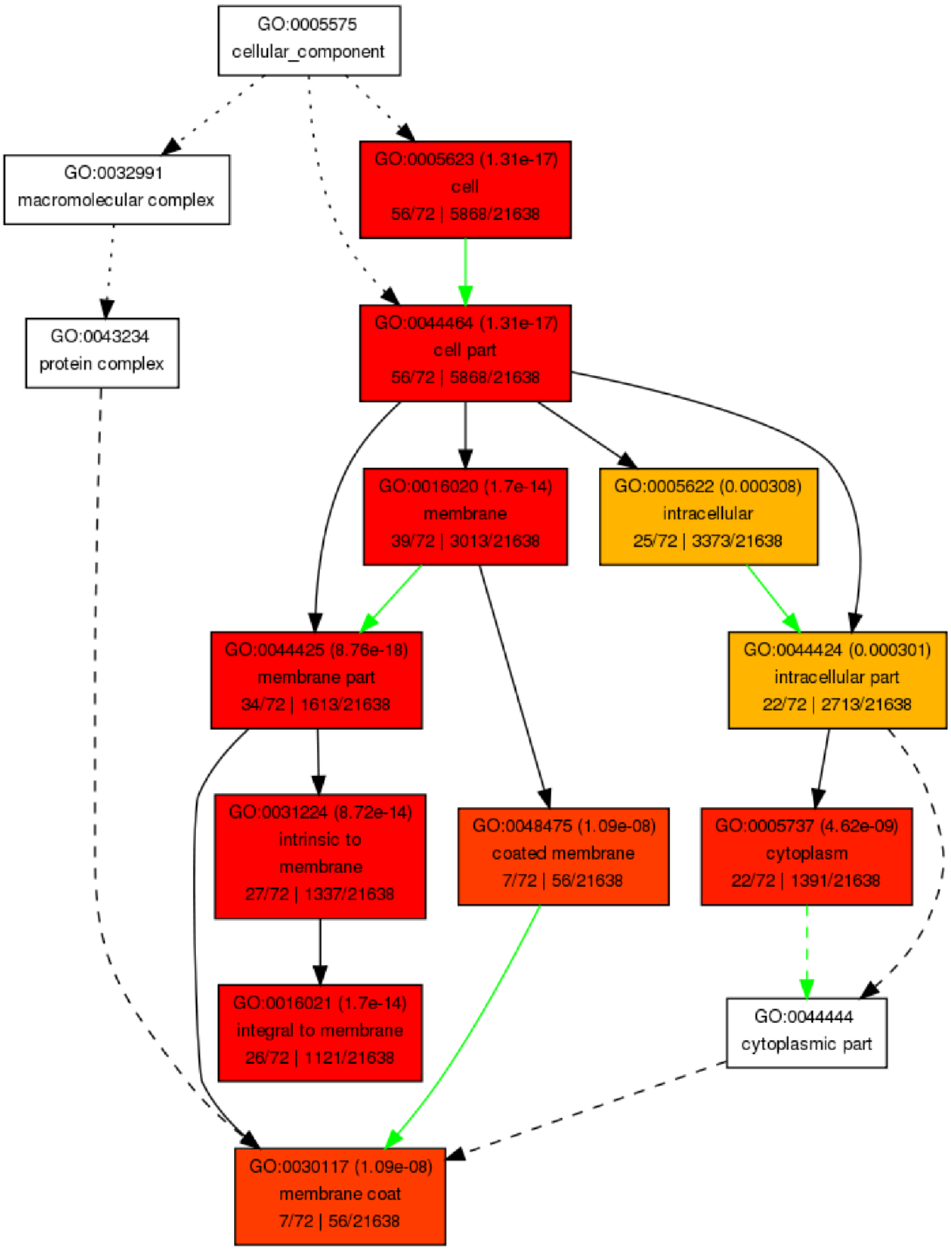

Figure S12. Details of SNPs located in *PtNHXs*.

Position	Classification	Alternate	Reference	Substi-tution	Genotype (549) ./.	0/0	0/1	1/1	Located motif
PtNHX1	**Potri.005G045100**								
3308285	non synonymous coding	C	G	H544D		518	31		_
3308495	stop gained	A	C	G474*		548	1		_
3308545	non synonymous coding	A	G	T457I		548	1		_
3308549	non synonymous coding	C	T	M456V		548	1		_
3308600	non synonymous coding	C	G	Q439E	1	546	2		_
3308618	non synonymous coding	A	C	V433L		547	2		TM10
3309434	non synonymous coding	C	G	L352V		542	5	2	TM8
3309440	non synonymous coding	A	C	G350C		541	8		TM8
3309443	non synonymous coding	C	G	L349V		548	1		TM8
3310054	non synonymous coding	G	T	M267L		548	1		_
3310079	non synonymous coding	C	T	I258M		547	2		_
3310845	non synonymous coding	A	G	R216C		548	1		_
3310847	non synonymous coding	G	A	V215A		548	1		_
3310989	non synonymous coding	T	G	L168I		548	1		_
3311082	intron splice site donor	CCAC	CCACAC			546	3		_
3312428	non synonymous coding	C	T	E44G		548	1		_
3312456	non synonymous coding	A	C	A35S		548	1		TM1
3312534	non synonymous coding	A	C	G9C		548	1		_
3312593	start gained	G	A			548	1		_
3313355	start gained	A	G		1	30	170	348	_
3313358	start gained	C	A			520	27	2	_
3313394	start gained	T	G			433	106	10	_
PtNHX2	**Potri.013G031700**								
2099568	non synonymous coding	A	T	Y529F	1	546	2		_
2099610	non synonymous coding	C	G	A515G		545	4		_
2099611	non synonymous coding	T	C	A515T	1	532	16		_
2099614	non synonymous coding	C	T	N514D	1	546	2		_
2099697	non synonymous coding	C	T	H486R		547	2		_
2099770	non synonymous coding	G	C	A462P		547	2		_
2100368	non synonymous coding	C	T	N412S		548	1		_
2101155	non synonymous coding	T	C	V300I		548	1		_
2101374	non synonymous coding	A	G	L263F		548	1		_
2102198	non synonymous coding	A	C	G233V		548	1		TM5
2102363	non synonymous coding	A	C	E213D		547	2		_
2102377	non synonymous coding	T	C	V209I		543	6		_
2102406	non synonymous coding	C	T	Q199R		548	1		_
2102497	non synonymous coding	T	C	G169S		547	2		_
2103426	non synonymous coding	A	T	Q105H		548	1		_
2104173	non synonymous coding	A	G	T57I		547	2		TM2
2104305	non synonymous coding	C	T	Q13R		543	6		_
2105061	start gained	C	A			548	1		_
2105153	start gained	C	G			546	3		_
PtNHX3	**Potri.013G026600**								
1756634	start lost	G	A	M1V		548	1		_

Figure S12 continue

1756643	non synonymous coding	C	G	E4Q		219	241	89	_
1756644	non synonymous coding	T	A	E4V		76	227	246	_
1756670	non synonymous coding	C	A	N13H		548	1		_
1756683	non synonymous coding	G	T	F17C		247	226	76	_
1756704	non synonymous coding	T	C	S24L		548	1		_
1756749	non synonymous coding	T	A	H39L		548	1		_
1756755	non synonymous coding	G	A	H41R		547	2		_
1756783	non synonymous coding	G	C	F50L		105	255	189	_
1756824	non synonymous coding	T	C	A64V		529	20		TM1
1756868	non synonymous coding	A	C	H79N		547	2		_
1756901	non synonymous coding	A	C	L90I		546	3		TM2
1757044	non synonymous coding	A	G	V102I	1	547	1		TM2
1757057	non synonymous coding	A	C	A106E		548	1		_
1757602	non synonymous coding	A	G	E175K		547	2		_
1757871	non synonymous coding	G	A	E205G		547	2		_
1757891	non synonymous coding	G	A	I212V		548	1		_
1757907	non synonymous coding	C	G	G217A		548	1		_
1757967	non synonymous coding	G	A	H237R		548	1		_
1757985	non synonymous coding	T	C	T243I		468	79	2	_
1758025	non synonymous coding	C	A	L256F		190	254	105	TM5
1759213	non synonymous coding	G	C	H325Q		548	1		_
1759451	non synonymous coding	G	A	R365G		548	1		_
1759452	non synonymous coding	C	G	R365T		547	2		_
1759791	non synonymous coding	G	A	N398S		548	1		_
1759816	non synonymous coding	A	C	S406R		547	2		_
1760383	non synonymous coding	G	C	T490S	1	547	1		_
1760405	non synonymous coding	G	C	D497E	2	546	1		_
1760409	non synonymous coding	G	A	R499G	1	547	1		_
1760421	non synonymous coding	T	C	L503F	2	544	3		_
1760495	non synonymous coding	G	A	I527M		546	3		_
1760498	non synonymous coding	T	A	R528S	1	543	5		_
1760587	non synonymous coding	A	T	F558Y		548	1		_
PtNHX4	**Potri.010G031500**								
4632425	non synonymous coding	A	G	A53T		519	30		TM2
4632589	non synonymous coding	T	A	L80F		548	1		TM3
4632962	non synonymous coding	G	T	H110M		450	94	5	TM4
4633157	non synonymous coding	A	G	R141K		548	1		_
4633440	non synonymous coding	T	G	E168D		548	1		_
4633553	non synonymous coding	A	T	I206N		548	1		_
4634108	non synonymous coding	A	C	S245Y	1	547	1		_
4634859	non synonymous coding	G	A	K368R		435	108	6	_
4634867	non synonymous coding	A	T	Y371N		548	1		_
4636205	non synonymous coding	T	G	R511L	2	546	1		_
PtNHX5	**Potri.014G134900**								
10266176	start gained	T	A		2	531	15	1	_
10266206	start gained	G	T		2	546	1		_
10266279	start gained	T	C			548	1		_
10266468	splice site acceptor	C	A			548	1		_
10266500	start lost	T	G	M1I	1	546	2		_

Figure S12 continue

10266507	non synonymous coding	T	G	G4C		548	1		_
10266543	non synonymous coding	G	A	I16V	3	542	4		TM1
10266558	non synonymous coding	A	T	L21M	1	541	7		TM1
10266862	non synonymous coding	A	C	P85H		548	1		TM3
10266882	intron:5 splice site donor:5	C	T			544	5		_
10267009	non synonymous coding	C	T	M107T		487	60	2	TM4
10267045	non synonymous coding	C	T	I119T		548	1		TM4
10267585	non synonymous coding	G	A	T125A		498	50	1	_
10267592	non synonymous coding	T	A	Q127L		538	11		_
10267615	non synonymous coding	C	G	G135R		538	11		_
10268159	non synonymous coding	T	C	T183M	2	546	1		_
10268164	non synonymous coding	G	A	I185V	2	424	114	9	_
10268281	non synonymous coding	A	G	A224T	1	547	1		TM5
10268769	non synonymous coding	G	A	I228V		548	1		TM5
10268770	non synonymous coding	C	T	I228T		548	1		TM5
10269307	non synonymous coding	C	G	W284S	1	547	1		TM6
10269319	non synonymous coding	A	C	S288Y		548	1		_
10269477	non synonymous coding	T	A	K296N		473	71	5	_
10269827	non synonymous coding	T	G	G343W		548	1		TM8
10270031	non synonymous coding	G	A	M385V		548	1		TM9
10271523	non synonymous coding	A	G	V480M	1	520	27	1	_
10271544	non synonymous coding	G	A	T487A	1	546	2		_
10271573	non synonymous coding	C	G	L496F	1	425	113	10	_
10271656	non synonymous coding	T	A	E524V		490	58	1	_
10271661	non synonymous coding	G	A	I526V		547	2		_
PtNHX6	**Potri.016G000200**								
14497	start gained	T	C			548	1		_
15600	non synonymous coding	A	G	R157K		548	1		_
17756	non synonymous coding	T	A	K217N		546	3		_
17790	non synonymous coding	A	G	V229I		548	1		_
18802	non synonymous coding	T	A	Y306F		458	83	8	_
19006	non synonymous coding	T	C	A341V	1	547	1		_
19403	non synonymous coding	A	G	R383H		548	1		_
21635	codon deletion	AAAT	AAATAAT	N467-	1	547	1		_
PtNHX7	**Potri.008G140700**								
9390553	start gained	G	A			548	1		_
9390657	non synonymous coding	G	A	I5M		544	5		_
9390665	non synonymous coding	A	G	G8E	1	547	1		_
9390686	non synonymous coding	C	T	I15T		548	1		_
9390713	non synonymous coding	T	C	A24V	1	547	1		_
9390793	non synonymous coding	G	T	L51V		548	1		TM1
9390838	non synonymous coding	A	G	V66I		524	22	3	TM2
9391131	non synonymous coding	G	T	V102G		542	7		TM3
9391763	non synonymous coding	A	C	L129I	2	546	1		TM4
9391784	non synonymous coding	G	C	L136V		532	17		TM4
9391929	intron:2 splice site acceptor:2	C	G			548	1		_
9392295	non synonymous coding	A	G	R211Q		543	5	1	_

Figure S12 continue

9392311	non synonymous coding	T	G	E216D		335	179	35	_
9392313	non synonymous coding	A	G	S217N		548	1		_
9392319	non synonymous coding	A	C	T219N		547	2		_
9392484	frame shift	TCC	TC	-250?	6	523	20		TM5
9392486	non synonymous coding	C	G	G250R	9	411	129		TM5
9392489	non synonymous coding	G	T	F251V	7	513	29		TM5
9392535	non synonymous coding	G	T	V266G	1	546	2		Additional TM2
9392543	non synonymous coding	A	G	V269I		344	174	31	Additional TM2
9393551	non synonymous coding	T	G	G290V		548	1		TM6
9393635	non synonymous coding	T	C	A294V		532	17		TM6
9393674	non synonymous coding	G	A	Q307R	1	454	88	6	_
9394638	non synonymous coding	T	C	A320V		548	1		TM7
9395547	non synonymous coding	G	A	T342A	1	547	1		_
9395557	non synonymous coding	A	G	S345N		548	1		_
9395561	non synonymous coding	A	T	H346Q		548	1		_
9397186	non synonymous coding	C	A	Y357S		548	1		TM8
9397218	non synonymous coding	A	G	V368I		548	1		TM8
9397251	non synonymous coding	T	G	G379C		548	1		_
9402781	non synonymous coding	T	A	I463L		548	1		_
9402806	non synonymous coding	C	T	M471T		548	1		_
9402833	non synonymous coding	T	G	G480V		548	1		_
9402848	non synonymous coding	G	A	D485G		548	1		_
9402937	non synonymous coding	A	G	G515S		548	1		_
9404147	non synonymous coding	T	A	Q554L		344	174	31	_
9404204	non synonymous coding	T	A	H573L		548	1		_
9404240	non synonymous coding	T	A	N585I	1	547	1		_
9406794	non synonymous coding	G	A	I646M		548	1		_
9407050	non synonymous coding	A	G	R674H		547	2		_
9407098	non synonymous coding	G	T	I690S		548	1		_
9407319	non synonymous coding	C	A	H731P	1	342	174	32	_
9407343	non synonymous coding	T	C	A739V	2	546	1		_
9407378	non synonymous coding	T	C	R751C	2	540	7		_
9407439	non synonymous coding	G	A	K771R	1	547	1		_
9407458	non synonymous coding	A	T	N777K		343	174	32	_
9407471	non synonymous coding	C	A	I782L	1	541	7		_
9407605	non synonymous coding	C	A	S791R	3	342	173	31	_
9407654	non synonymous coding	A	G	G807E	1	547	1		_
9407712	non synonymous coding	G	A	I826M	1	547	1		_
9407716	non synonymous coding	T	G	D828Y	1	546	2		_
9407729	non synonymous coding	A	T	L832H		343	175	31	_
9407738	non synonymous coding	G	T	F835C		548	1		_
9407758	frame shift	CT	CTTT	-842		548	1		_
9407773	non synonymous coding	G	T	S847A		548	1		_
9407807	intron:2 splice site donor:2	G	T		1	413	135		_
9408180	non synonymous coding	T	C	P902L	1	343	175	30	_
9408246	non synonymous coding	T	C	T924I		346	172	31	_

Figure S12 continue

9408251	non synonymous coding	C	T	S926P		351	167	31	_
9409911	non synonymous coding	T	G	D971Y	1	546	2		_
9409932	non synonymous coding	A	G	D978N	2	546	1		_
9409950	non synonymous coding	G	A	R984G		547	2		_
9409951	non synonymous coding	T	G	R984I		344	173	32	_
9409969	non synonymous coding	T	C	S990L		547	2		_
9410004	non synonymous coding	T	C	H1002Y		548	1		_
9410044	non synonymous coding	A	C	P1015Q		547	2		_
9410053	non synonymous coding	T	G	R1018L		515	31	3	_
9410073	non synonymous coding	C	A	T1025P	1	547	1		_
9410096	non synonymous coding	C	G	L1032F	1	547	1		_
9410124	non synonymous coding	A	T	F1042I	1	547	1		_
9410457	non synonymous coding	A	G	M1048I	1	545	3		_
9410482	non synonymous coding	A	G	G1057S		526	20	3	_
9410497	stop gained	T	C	R1062*	1	546	2		_
9410531	non synonymous coding	T	C	S1073L		548	1		_
9410612	non synonymous coding	T	C	T1100I	1	229	242	77	_
9410659	non synonymous coding	G	A	I1116V		548	1		_
9410725	non synonymous coding	T	A	R1138W		539	10		_
PtNHX8	**Potri.010G100900**								
12220655	non synonymous coding	C	T	S1139G		548	1		_
12220716	non synonymous coding	T	C	M1118I		548	1		_
12220723	non synonymous coding	C	T	H1116R	1	544	4		_
12220727	non synonymous coding	A	T	T1115S		548	1		_
12220731	non synonymous coding	T	A	N1113K		548	1		_
12220789	non synonymous coding	A	T	E1094V		548	1		_
12220793	non synonymous coding	T	G	L1093I		548	1		_
12220795	non synonymous coding	G	C	S1092T		547	2		_
12220811	non synonymous coding	C	T	T1087A		104	257	188	_
12220844	non synonymous coding	C	G	R1076G	2	390	140	17	_
12220913	non synonymous coding	C	T	S1053G		547	2		_
12221328	non synonymous coding	T	A	C1021S		444	95	10	_
12221439	non synonymous coding	A	G	P984S		547	2		_
12221456	non synonymous coding	T	C	G978D		548	1		_
12221506	non synonymous coding	A	C	E961D	1	547	1		_
12221537	non synonymous coding	G	A	F951S	1	547	1		_
12223079	non synonymous coding	C	T	I943M		548	1		_
12223107	non synonymous coding	A	C	G934V		548	1		_
12223140	non synonymous coding	G	A	I923T		548	1		_
12223249	non synonymous coding	C	T	R887G	1	547	1		_
12223251	non synonymous coding	C	T	Q886R	1	393	142	13	_
12223279	non synonymous coding	T	A	L877M		548	1		_
12223282	non synonymous coding	A	G	P876S		548	1		_
12223285	non synonymous coding	A	T	M875L		548	1		_
12223566	intron splice site donor	C	A		7	440	102		_
12223591	non synonymous coding	T	C	A850T		548	1		_
12223714	non synonymous coding	C	T	T809A		546	3		_
12223744	non synonymous coding	A	C	A799S		547	2		_
12223899	non synonymous coding	A	C	V786F	1	547	1		_

Figure S12 continue

12223932	non synonymous coding	C	T	K775E	1	547	1		_
12223955	non synonymous coding	G	A	V767A	1	547	1		_
12224226	non synonymous coding	G	C	K705N		548	1		_
12224264	non synonymous coding	T	C	V693I		529	19	1	_
12224272	non synonymous coding	G	A	I690T		548	1		_
12228138	non synonymous coding	T	C	R632Q		446	94	9	_
12228251	non synonymous coding	T	G	F594L	1	544	4		_
12228328	non synonymous coding	G	C	D569H		548	1		_
12228331	non synonymous coding	A	T	I568F		444	94	11	_
12228409	non synonymous coding	T	C	A542T		539	9	1	_
12229610	non synonymous coding	T	G	R534S		548	1		_
12229613	non synonymous coding	T	A	L533I		548	1		_
12229630	non synonymous coding	G	T	N527T		548	1		_
12229654	non synonymous coding	A	G	A519V		548	1		_
12229670	non synonymous coding	A	G	H514Y		547	2		_
12229694	non synonymous coding	T	A	L506M		548	1		_
12229727	non synonymous coding	A	T	T495S	1	547	1		_
12229811	non synonymous coding	A	T	T467S		548	1		_
12230379	non synonymous coding	A	G	A458V		532	17		_
12230452	non synonymous coding	T	G	L434I		548	1		TM10
12230464	non synonymous coding	A	C	G430C		548	1		TM10
12231126	non synonymous coding	G	C	A398P	1	547	1		TM9
12231137	non synonymous coding	G	C	G394A	1	547	1		TM9
12231150	non synonymous coding	T	G	L390I	1	520	27	1	TM9
12231239	non synonymous coding	G	A	V360A		548	1		TM8
12231246	non synonymous coding	C	T	I358V		548	1		TM8
12231258	non synonymous coding	G	A	F354L		548	1		TM8
12235111	non synonymous coding	T	C	A300T		548	1		TM6
12235116	non synonymous coding	G	C	R298T		547	2		TM6
12235253	non synonymous coding	A	C	E276D		546	3		_
12236291	non synonymous coding	C	A	F251V	6	398	145		TM5
12236294	non synonymous coding	G	C	G250R	6	524	19		TM5
12236434	non synonymous coding	A	G	T228I		544	5		_
12236456	non synonymous coding	A	C	V221F		548	1		_
12236515	non synonymous coding	A	G	T201I		548	1		_
12236717	non synonymous coding	T	G	S195Y	1	545	3		_
12236780	non synonymous coding	G	A	V174A		547	2		Additional TM1
12236837	non synonymous coding	C	T	N155S		547	2		_
12236969	non synonymous coding	C	T	I137V		495	51	3	TM4
12237535	non synonymous coding	C	A	F109L		548	1		TM3
12237557	non synonymous coding	C	A	V102G		532	17		TM3
12237579	non synonymous coding	T	C	D95N	1	441	98	9	_
12237699	non synonymous coding	C	T	D86G	2	395	136	16	_
12237904	non synonymous coding	T	C	R49K	1	546	2		TM1
12238012	non synonymous coding	C	T	Y13C		548	1		_
12238085	start gained	C	T			548	1		_

Supplementary Tables:

Table S1. List of NHX genes and their coding amino acid sequences identified from *P. trichocarpa, E. grandis, V. vinifera, A. thaliana, M. truncatula, G. max, O. sativa, S. bicolor, B. distachyon, Z. mays,* and *P. patens*.

Table S2. The sequences of qRT-PCR primers.

Table S3. GO enrichment analysis of genes co-expressed with *AtNHXs*.

Figure S1. Nucleotide and amino acid sequences of selected three *PtNHXs* (*-1*, *-6*, and *-8*) from three major groups cloned from *P. trichocarpa*.

The three cloned *PtNHXs* (*-1*, *-6*, and *-8*) showed 99.45%, 99.57%, and 99.54% identities with their corresponding CDS sequences from the *P. trichocarpa* genome database, respectively.

Figure S2. *Arabidopsis NHX* map position and duplication analysis.

(A) Schematic representation of *Arabidopsis* chromosomes showing the location of *AtNHX* genes. **(B)** The duplicated *AtNHX* genes (red labelled) on *Arabidopsis* chromosomes.

Figure S3. Sliding window analysis of duplicated *PtNHXs*.

The window size 60 and 90 bp were used for *K*a/*K*s analysis. The sequence identities and TM regions were shown in bottom of each figure.

Figure S4. Details of motifs in AtNHXs and PtNHXs identified by MEME.

Figure S5. Multiple sequence alignment and transmembrane region of PtNHXs.

Figure S6. Details of secondary structure of PtNHX proteins.

Figure S7. Expression patterns of *Arabidopsis NHX* genes across different tissues.

The data was obtained from AtGenExpress Visualization Tool (http://weigelworld.org/resources).

Figure S8. Expression patterns of *Arabidopsis NHX* genes under various abiotic stresses.

The microarray data was obtained from AtGenExpress Visualization Tool. Red means highly expression and green indicates low expression.

Figure S9. GO enrichment analysis of genes co-expressed with *PtNHXs*

Figure S10. Co-expression network of *Arabidopsis NHX* genes.

Figure S11. GO enrichment analysis of PtNHX PPI network.

Figure S12. Details of SNPs located in *PtNHXs*.

Table S1. List of NHX genes and their coding amino acid sequences identified from *P. trichocarpa, E. grandis, V. vinifera, A. thaliana, M. truncatula, G. max, O. sativa, S. bicolor, B. distachyon, Z. mays,* and *P. patens*.

Table S2. The sequences of qRT-PCR primers.

Table S3. GO enrichment analysis of genes co-expressed with *AtNHXs*.
